# Interplay between C-reactive protein responses and antibiotic prescribing in people with suspected infection

**DOI:** 10.1186/s12879-025-11381-9

**Published:** 2025-08-05

**Authors:** Qingze Gu, Kevin Yuan, Jia Wei, Chang Ho Yoon, Anders Skyrud Danielsen, Augustine Luk, David W. Eyre, A. Sarah Walker

**Affiliations:** 1 Nuffield Department of Medicine, University of Oxford, Oxford, UK; 2 Nuffield Department of Population Health, Big Data Institute, University of Oxford, Oxford, UK; 3 NIHR Health Protection Research Unit in Healthcare Associated Infections and Antimicrobial Resistance, University of Oxford, Oxford, UK; 4 NIHR Oxford Biomedical Research Centre, Oxford, UK; 5 Oxford University Hospitals NHS Foundation Trust, Oxford, UK; 6 Department of Microbiology, Oslo University Hospital, Oslo, Norway; 7 Department of Infection Control and Preparedness, Norwegian Institute of Public Health, Oslo, Norway

**Keywords:** Bloodstream infection, Antibiotic prescribing, C-reactive protein, Centile reference charts, Antibiotic stewardship, Antimicrobial resistance

## Abstract

**Background:**

Serial measurements of C-reactive protein (CRP) are often taken in hospitals to assess recovery from infection, but their utility remains debated. Previous studies, including our development of CRP centile reference charts for suspected bloodstream infections (BSI), suggest variability in CRP responses across infection types. Here we investigated the association between serial CRP percentile changes, antibiotic prescribing patterns, and patient outcomes in a large cohort with suspected infection, acknowledging that CRP is one of multiple factors in clinical decision-making.

**Methods:**

We analysed 51,544 suspected infection episodes (defined by blood culture collection) from 36,578 patients in Oxfordshire, UK (2016–2021). Episodes were categorised by blood culture results: Gram-positive, Gram-negative, polymicrobial, contaminants, or culture-negative (having previously shown that 51% culture-negatives have CRP responses indistinguishable from culture-positives). The spectrum of antibiotic prescriptions and their changes over time were tracked. Multinomial logistic regression, adjusted for clinical covariates, assessed the association between CRP percentile changes and subsequent prescribing decisions. Linear mixed models evaluated CRP trajectories post-prescribing, and logistic regression associations between early CRP changes (days 1–4) and 5–30-day mortality.

**Results:**

Broad-spectrum antibiotics were predominantly used within the first three days after blood culture collection, followed by a notable shift to narrow-spectrum antibiotics for Gram-positive infections, but with slower de-escalation for Gram-negative and polymicrobial infections. CRP percentile changes were modestly associated with subsequent antibiotic adjustments; in particular, suboptimal recovery, indicated by an increase in CRP centiles, was associated with a higher rate of antibiotic escalation (16.5% vs. 10.7% in expected recovery) and, conversely, faster than expected recovery in CRP was associated with de-escalation (23.6% vs. 17.2%). However, 61.8% of decisions were unchanged despite CRP trends. The relationship between various prescribing decisions and subsequent CRP percentile changes was complex and challenging to estimate, likely due to testing bias. CRP percentile changes during the 4 days post blood culture collection were strongly associated with 5–30-day mortality, highlighting their potential utility as a prognostic indicator.

**Conclusions:**

While CRP monitoring can inform antibiotic stewardship, its association with prescribing decisions is probably only modest, underscoring the need to integrate a range of clinical factors to optimise infection management.

**Supplementary Information:**

The online version contains supplementary material available at 10.1186/s12879-025-11381-9.

## Introduction

Suspected bloodstream infections (BSI) remain an important challenge in clinical practice due to their high morbidity and mortality rates [1]. Effective management necessitates the prompt initiation of empirical antibiotic therapy before a definitive microbiological diagnosis is available [2]; further, no pathogen is ever cultured in the majority [3–5]. While essential, this approach carries inherent risks, including the possible lack of efficacy of inappropriate narrow-spectrum coverage or the potential for fostering antimicrobial resistance through the overuse of broad-spectrum antibiotics [6]. Given these concerns, optimising antibiotic prescribing practices is of paramount importance.

A crucial aspect of infection management involves the timely adjustment of antibiotic therapy based on patient-specific indicators. Empirically, broad-spectrum antibiotics are often prescribed due to the high consequences of under-treatment in the early stages of suspected serious infection [7]. However, this must be balanced with the need to de-escalate to narrower-spectrum agents as soon as pathogen-specific antibiotic susceptibilities are identified or clinical markers suggest a favourable response [6, 8–10]. Strategic de-escalation not only mitigates resistance development but also minimises unwanted side effects, supporting the overall goals of antibiotic stewardship [10, 11].

C-reactive protein (CRP) is one of many tools in a multifactorial decision-making process that includes vital signs, microbiological results, imaging, and clinical judgment. Serial CRP measurements are frequently requested by clinicians aiming to assess recovery from infections and tailor treatment accordingly, including in BSI [12, 13]. However, the evidence to support their use is mixed. Changes in CRP levels within the first 4–5 days can indicate whether patients are responding adequately to treatment or modifications are required [14, 15], with decreasing CRP levels generally indicating effective control of infection, while non-decreasing or increasing levels signalling treatment failure or complications [14–16]. Typically, studies have considered the percentage drop from the observed peak [17–19], whether absolute levels fall below a pre-specified threshold [17, 19], or the ratio of CRP measurements on days 4 or 5 relative to baseline levels [16, 20, 21]. However, others have questioned the diagnostic accuracy of CRP for individual patient use, even labelling them ‘zombie tests’ [22].

**Fig. 1:**
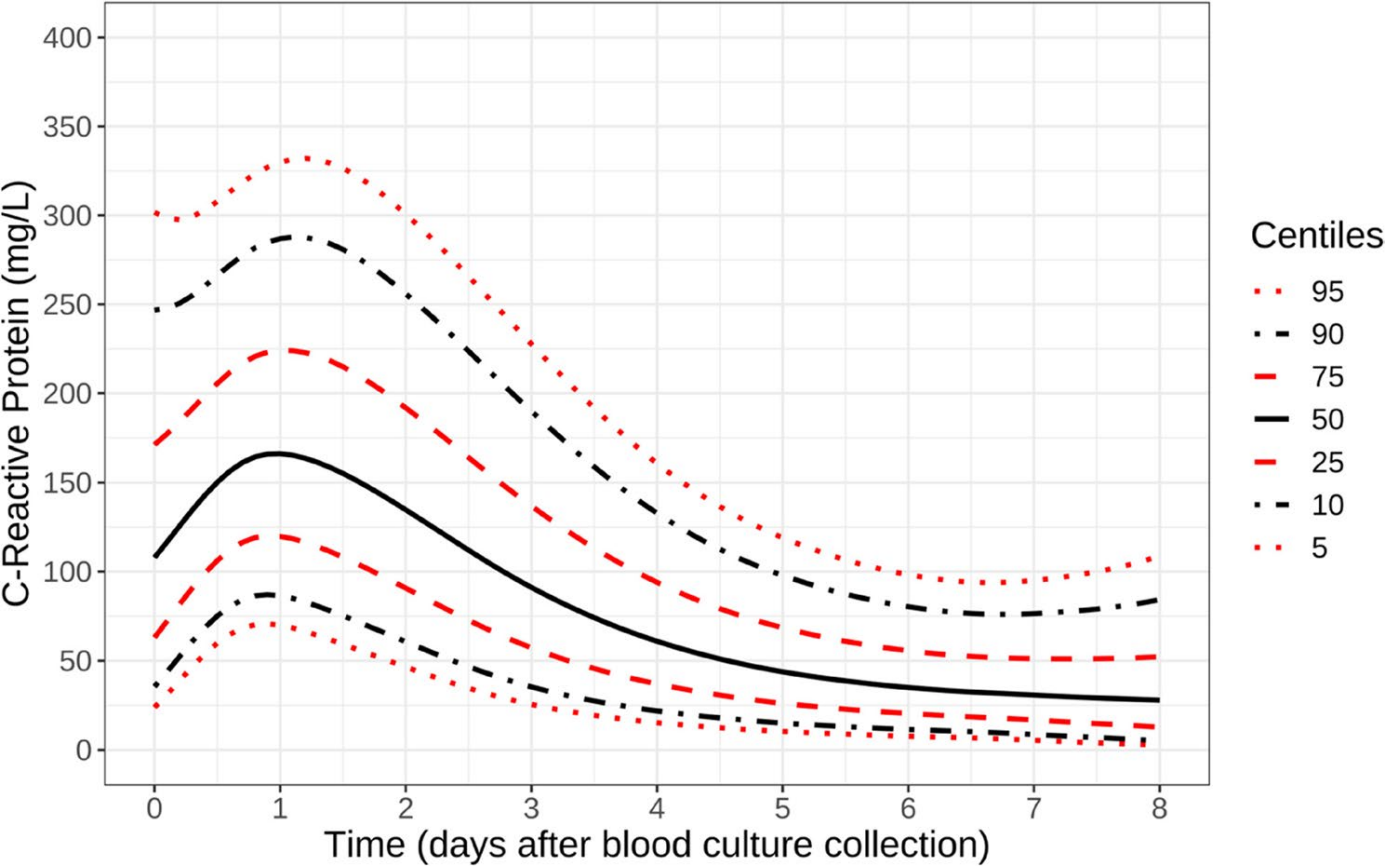
Centile reference chart of expected CRP responses in patients with culture-positive/negative suspected BSI responding standardly to antimicrobials. Note: estimated from standard responders with peak CRP response on day 1/2, regardless of pathogen isolated. Reproduced under the terms of the CC-BY licence agreement from [5]

We recently developed centile reference charts for expected CRP and vital sign trajectories during suspected BSI episodes, analogous to paediatric growth charts, providing a potential tool to monitor patients’ recoveries [5] (Fig. 1). A centile-based approach is used to account for heterogeneity in initial CRP responses arising from different clinical syndromes, pathogens, and host factors to provide a simple single chart for tracking patient responses. Patients who recover as expected track along the percentile line on which they first started, while patients not recovering as expected change CRP centiles. Moreover, we showed that 51% of patients with culture-negative suspected infection episodes nevertheless had CRP responses which were indistinguishable from those with culture-positive infection [5], highlighting the fact that many of these episodes are likely to be genuine infection where a pathogen was not detected, for example because insufficient amount of blood cultured or the infection was localised rather than in the blood stream [23–25], or because prior antibiotics prevented blood cultures from being positive.

Here we therefore examined the association between the dynamics of CRP percentile changes and antibiotic prescribing in a large cohort of patients with suspected infection, as indicated by blood being taken for culture, to investigate further their potential use in targeting and adjusting empiric antibiotic therapy. We aimed to: first, investigate the pattern of antibiotic prescription over time in suspected infections; second, elucidate whether and how changes in CRP centiles are associated with subsequent prescribing behaviours; and third, evaluate the relationship between changes in antibiotics and subsequent CRP centiles.

## Methods

### Study population

We used de-identified data from the Infections in Oxfordshire Research Database, containing information from all inpatient admissions at the Oxford University Hospitals NHS Foundation Trust (OUH), United Kingdom, together with vital signs, microbiology and biochemistry/haematology results and antimicrobials prescribed in hospital (including at discharge). OUH contains ∼1100 beds in four hospitals, providing all acute care and pathology services to a population of ∼750,000 and specialist services to the surrounding region.

Patients ≥ 16 years old who had at least one blood culture during an inpatient admission between 1-January-2016 and 28-June-2021 were included. We included all episodes in which one or more blood cultures were obtained, using the culture collection as a pragmatic indicator that clinicians suspected a serious bloodstream or systemic infection. This definition captures both culture-positive and culture-negative presentations, reflecting everyday practice in which only 30–40% of suspected cases yield a bloodstream pathogen [3–5]. Blood culture isolates were classified as Gram-positive, Gram-negative and other pathogens, or potential contaminants and culture negative (See Table S1 for pathogen distribution). We defined a new suspected infection episode when there were > 14 days since the last collection of blood for culture using the culture dataset containing pseudonymised patient identifier, date and time of blood sample collection, and blood culture results, as described previously [5]. Where more than one set of blood cultures were obtained within a suspected infection episode, we prioritised pathogens, then contaminants, then any negative cultures as the index blood culture defining each episode’s start (date/time of the blood collection for culture).

We excluded infection episodes where fungi were detected in blood cultures because the current analysis focused on bacterial infection and antibiotic treatments (Figure S1). After merging with information about inpatient admissions and lab measurements, we excluded episodes if patients were admitted directly to ICU (where antimicrobial prescriptions were unknown because these were recorded on different systems), lacked baseline vital signs data, or had no CRP measurements or no antibiotic administration records (Figure S1).

## Definitions

### Antibiotic hierarchy and escalation/de-escalation

Antibiotics were grouped and ranked with reference to existing research [26], considering the spectrum of activity and clinical importance, and adapted to the antibiotics available and prescribing practices in our hospital, given our goal was to investigate associations between CRP decisions and prescribing which was undertaken within this local context (**Table S2**). This classification resulted in five groups, ranging from Group 1, representing the most narrow-spectrum antibiotics, to Group 5, representing the most broad-spectrum antibiotics. Antibiotic differences between two timepoints were defined as “escalation”, “unchanged”, “de-escalation”, or “stop”, based on differences in this pre-defined antibiotic spectrum ranking, the number of different antibiotics administered, and the route of administration. These definitions first considered antibiotic spectrum (**Table S2**): a move to a higher-ranked group was “escalation”, and to a lower-ranked group was “de-escalation”. If the spectrum ranking of the highest-ranked antibiotic remained the same, other factors were then assessed; specifically a switch from intravenous to oral administration, or a reduction in the number of concurrently administered antibiotics, was classified as “de-escalation”, and vice versa for “escalation”. When multiple antibiotics were used at a given point in time, the highest-ranked antibiotic determined the antibiotic ranking; intravenous was dominant if both intravenous and oral were used. Stopping was defined as ≥ 48 h between successive doses of antibiotics or after the last recorded dose was administered; if the patient died within 14 days of the last dose, it was defined as “stopped and died within 14d”.

### Covariates

We defined community-onset infections as those where the index blood culture was taken ≤ 48 h after admission. Charlson comorbidity and Elixhauser acuity scores were calculated using ICD-10 diagnostic codes based on a 1-year lookback period, adding all primary and secondary ICD-10 codes in the year prior to the inpatient episode containing the index blood culture to all secondary ICD-10 codes from the current inpatient episode [27]. An additional covariate, immunosuppression, was determined by the presence of ICD-10 diagnostic codes for AIDS/HIV (B20–24), metastatic cancer and haematological malignancies (C77-96), primary immunodeficiencies (D80-84) and end-stage liver disease (K721, K729, K766, K767) within the same 1-year lookback period. Similarly, palliative care (Z515), diabetes mellitus with/without complications (E100-149) and end-stage renal disease requiring dialysis (Z992, Z49, N186, T824) were determined by the presence of relevant ICD-10 diagnostic codes within the same 1-year lookback period. Infection sources were identified from free text antimicrobial prescribing indications within 1 day before to 8 days after the start of each episode using a pre-developed natural language processing model [28]. The infection sources were categorised into respiratory, urinary, abdominal, skin/soft tissue/orthopaedic, central nervous system (CNS), other, multiple sources, and unspecific, based on the antimicrobial prescribing indications. The ‘unspecific’ category included episodes where the prescribing indication did not specify a particular infection source, while ‘other’ included less common sources not classified into the main categories. Baseline NEWS (National Early Warning Score, specifically NEWS2) was calculated using the vital signs recorded closest to the time of index blood culture collection (within 24 h before or after). NEWS2 is a composite score derived from six physiological parameters: respiratory rate, oxygen saturation, use of supplemental oxygen, systolic blood pressure, pulse rate, level of consciousness (AVPU scale), and temperature. Specific scores (ranging from 0 to 3) are assigned to different measurement ranges for each parameter, and these scores are then summed to produce the total NEWS2 value [29].

## Research design and statistical analyses

### Antibiotic prescribing

Records of antibiotic treatment prescribed in the hospital (both in-hospital and discharge prescriptions) within 14 days following index blood culture collection were grouped, and antibiotics were ranked using the antibiotic spectrum ranking above (Table S2).

### CRP percentile changes and subsequent antibiotic prescribing

CRP measurements and antibiotic treatment records within 8 days after taking index blood cultures were included in analyses. Absolute CRP levels were converted to centiles based on the pre-defined centile chart [5]. Multinomial logistic regression was used to investigate the association between subsequent prescribing decisions -classified as escalation, unchanged (including no change in prescription or a change but with the same antibiotic ranking, number of drugs and route), de-escalation, or stop - and prior CRP percentile changes.

We compared antibiotics given in the 24 h after the second of two consecutive daily CRP measurements with those given at the time of the second CRP measurement (Figure S2; see Figure S1 for exclusion criteria). CRP percentile change was truncated at ± 30 centiles, and potential non-linearity in its effect on antibiotics received was modelled using natural cubic splines with three knots at −15, 0, and 15 (based on prior findings [5]), and boundary knots at −25 and 25, interacting with the timing of the subsequent prescribing decision relative to blood culture collection (interaction *p* < 0.0001), which was also modelled with natural cubic splines using three knots at days 3, 5, and 7, and boundary knots at days 2.2 and 8.1 (95% percentiles).

### Antibiotic prescribing changes and subsequent CRP percentile changes

Linear mixed models were used to estimate subsequent CRP percentile changes over time following different prescribing decisions, including all CRP measurements from a new prescribing decision up to 48 h after or until the next prescribing change, whichever occurred first (Figure S3; see Figure S1 for exclusion criteria). CRP measurements taken within 12 h before the new prescription were also included if CRP was measured afterwards. Sensitivity analysis included all CRP measurements from 12 h before a new prescribing decision up to 48 h after or until the next prescribing change and showed similar results (data not shown). Patients with only one CRP measurement after the prescribing decision were also included in this analysis, as they can still contribute population-level information about the average CRP percentile values in the study population [30]. The possibility that percentile trajectories varied non-linearly over time from the most recent prescribing decision was incorporated using natural cubic splines (as fixed and random (prescription-specific) effects) with one knot at 24 h, and boundary knots at −8.6 h and 43.4 h (95% percentiles). Models were adjusted (as fixed effects) for the type of prescribing decisions (escalation, unchanged, de-escalation, stop as above) and the day of prescribing relative to blood culture collection (as a natural cubic spline using three knots at days 1, 3, and 5, and boundary knots at days 0.1 and 6.4 (95% percentiles)). Two-way interactions between the type of prescribing decision, time following the prescribing decision, and the day of prescribing after blood culture collection were included (interaction *p* < 0.0001).

### The association between early percentile changes and 5–30 day all-cause mortality

Kaplan-Meier survival curves for mortality during 5–30 days after blood culture collection were computed and stratified by patient-level mean CRP percentile change per day calculated over days 1–4 (the post-peak recovery period based on prior findings [5]) using ordinary least squares (OLS) regression for each individual patient. Patients who died on or before day 4 (i.e., within the predictor assessment window) were excluded to ensure that the mortality outcome (occurring between days 5–30) was assessed only in patients who had survived the full period during which the predictor variable (mean CRP percentile change per day over days 1–4) was measured, thereby maintaining a clear temporal sequence between the predictor and the subsequent outcome. As there was no evidence of non-proportional hazards, very few patients were last seen alive before 30 days (i.e. were censored, 1.9% [244/13,141]), and in order to calculate AUROC, we then used logistic regression to estimate associations between 5 and 30 day all-cause mortality and individual patient-level mean percentile change per day between days 1–4 (treating censored patients as alive at day 30). Non-linearity was incorporated using natural cubic splines using five knots at −15, −7.5, 0, 7.5, and 15 and boundary knots at −25 and 25. AUROCs were calculated to evaluate the predictive power of various models for 5–30 day all-cause mortality, including covariates (see below) only, percentile changes, absolute CRP change, and log CRP change per day (days 1–4), and combinations of covariates with these.

All models for the three analyses described above were also adjusted for the following covariates as described previously to account for potential confounding factors [5]: infection source (identified from antimicrobial prescribing indications [28]), community-onset (≤ 48 h after admission), blood culture result (positive, potential contaminant, negative) and pathogen group (based on genus and clinical significance), age, sex, Charlson and Elixhauser scores, renal dialysis, diabetes mellitus, baseline NEWS, immunosuppression, and palliative care.

## Results

This analysis included 51,544 suspected infection episodes (defined by blood culture collection) in 36,578 patients presenting to hospitals in Oxfordshire, UK, between 1-January-2016 and 28-June-2021 [5]. We included suspected infections from patients who had both CRP measurements and were treated with antibiotics within 14 days following index blood culture collection (Table 1). A single Gram-positive pathogen was identified from blood cultures in 1,455 (2.8%) episodes, a single Gram-negative pathogen in 2,925 (5.7%), 775 (1.5%) had other pathogens or were polymicrobial, 2,646 (5.1%) had only a potential contaminant, and 43,743 (85.0%) were blood culture-negative. In previous analyses of this cohort [5], a substantial proportion of these culture-negative episodes were shown to have CRP responses indistinguishable from those observed in culture-positive episodes, suggesting many represent true infections. The median patient age was 71.0 (IQR 54.6–82.5) years. Among these episodes, 989 (1.9%) patients died ≤ 4 days after the index blood culture collection and were excluded from analyses of mortality between 5 and 30 days, while 4,223 (8.2%) died between 5 and 30 days.

**Table 1:** Characteristics at the start of 51,544 suspected infection episodes receiving antibiotic treatment between 01 January 2016 and 28 June 2021

Characteristic	Gram-positive pathogens, *N* = 1,455 (2.8%)^1^	Gram-negative pathogens, *N* = 2,925 (5.7%)^1^	Other pathogens/polymicrobial, *N* = 775 (1.5%)^1^	Potential contaminant(s), *N* = 2,646 (5.1%)^1^	Culture-negative, *N* = 43,742 (85%)^1^	Overall, *N* = 51,544 (100%)^1^
Age at admission (years)	72.5 (55.8, 83.4)	75.8 (63.4, 84.8)	66.4 (49.6, 80.3)	71.9 (56.7, 83.1)	70.6 (54.0, 82.2)	71.0 (54.6, 82.5)
Sex (male)	874 (60.1%)	1,637 (56.0%)	434 (56.0%)	1,345 (50.8%)	22,520 (51.5%)	26,810 (52.0%)
Charlson score	1 (1, 3)	2 (1, 3)	1 (1, 3)	2 (1, 3)	1 (0, 2)	1 (0, 2)
Elixhauser score	3 (2, 5)	3 (2, 5)	3 (1, 4)	3 (2, 5)	3 (1, 4)	3 (1, 4)
Renal dialysis	46 (3.2%)	62 (2.1%)	15 (1.9%)	40 (1.5%)	844 (1.9%)	1,007 (2.0%)
Diabetes mellitus	393 (27.0%)	779 (26.6%)	147 (19.0%)	620 (23.4%)	9,221 (21.1%)	11,160 (21.6%)
NEWS score (baseline)	3 (2, 6)	4 (2, 6)	3 (1, 5)	3 (1, 5)	3 (1, 5)	3 (1, 5)
Immunosuppression	245 (16.8%)	573 (19.6%)	212 (27.4%)	466 (17.6%)	7,161 (16.4%)	8,657 (16.8%)
Palliative care	135 (9.3%)	273 (9.3%)	96 (12.4%)	199 (7.5%)	2,732 (6.2%)	3,435 (6.7%)
Community-onset	1,161 (79.8%)	2,293 (78.4%)	567 (73.2%)	1,929 (72.9%)	35,679 (81.6%)	41,629 (80.8%)
Total blood cultures in episode	3 (2, 4)	2 (1, 3)	2 (1, 5)	2 (1, 3)	1 (1, 2)	1 (1, 2)
Vital status						
Died ≤ 4 days	41 (2.8%)	91 (3.1%)	20 (2.6%)	68 (2.6%)	769 (1.8%)	989 (1.9%)
Died 5–30 days	152 (10.4%)	272 (9.3%)	93 (12.0%)	230 (8.7%)	3,476 (7.9%)	4,223 (8.2%)
Survived > 30 days*	1,262 (86.7%)	2,562 (87.6%)	662 (85.4%)	2,348 (88.7%)	39,498 (90.3%)	46,332 (89.9%)
Source of infection						
Respiratory	347 (23.8%)	287 (9.8%)	117 (15.1%)	865 (32.7%)	14,987 (34.3%)	16,603 (32.2%)
Multiple sources	336 (23.1%)	689 (23.6%)	156 (20.1%)	454 (17.2%)	7,133 (16.3%)	8,768 (17.0%)
Urinary	119 (8.2%)	872 (29.8%)	75 (9.7%)	331 (12.5%)	5,618 (12.8%)	7,015 (13.6%)
Abdominal	76 (5.2%)	477 (16.3%)	114 (14.7%)	200 (7.6%)	4,398 (10.1%)	5,265 (10.2%)
Skin, soft tissue, orthopaedic	235 (16.2%)	65 (2.2%)	60 (7.7%)	198 (7.5%)	4,152 (9.5%)	4,710 (9.1%)
CNS	15 (1.0%)	15 (0.5%)	13 (1.7%)	32 (1.2%)	512 (1.2%)	587 (1.1%)
Other	88 (6.0%)	44 (1.5%)	64 (8.3%)	120 (4.5%)	1,054 (2.4%)	1,370 (2.7%)
Unspecific	239 (16.4%)	476 (16.3%)	176 (22.7%)	446 (16.9%)	5,889 (13.5%)	7,226 (14.0%)
^1^Median (IQR); n (%)						

Percentages in the header are of all episodes, and in the main body are column percentages within each group; continuous variables are summarised using the median (IQR). The baseline NEWS was calculated using the closest set of vital signs within 1 day before to 1 day after the start of each episode. *1.9% (244/13,141) patients were last seen alive before day 30 and were censored. See Table S1 for a full breakdown of microorganisms isolated from blood culture

### Antibiotic prescribing

The total durations of antibiotic treatment within 14 days post index blood culture, including antibiotics given as an inpatient and post-discharge, were (median (IQR)): Gram-positive infections 12.8 (7.5–13.9) days, Gram-negative infections 8.7 (6.6–13.4) days, other/polymicrobial infections 10.7 (6.6–13.9) days, suspected infections where only contaminants were isolated 6.8 (4.6–12.6) days, and suspected infections that were blood culture-negative 6.6 (4.6–11.3) days.

Reflecting underlying clinical syndromes and illness severity, the use of any empirical broad-spectrum antibiotics during days 1–3 post blood cultures (including broad-spectrum antibiotics, extended-spectrum antibiotics, anti-pseudomonal agents, and protected antibiotics), was more common in Gram-negative infections (2912/2,925, 99.6%) and polymicrobial infections (186/193, 96.4%), than in Gram-positive infections (1,262/1,455, 86.7%).

**Fig. 2:**
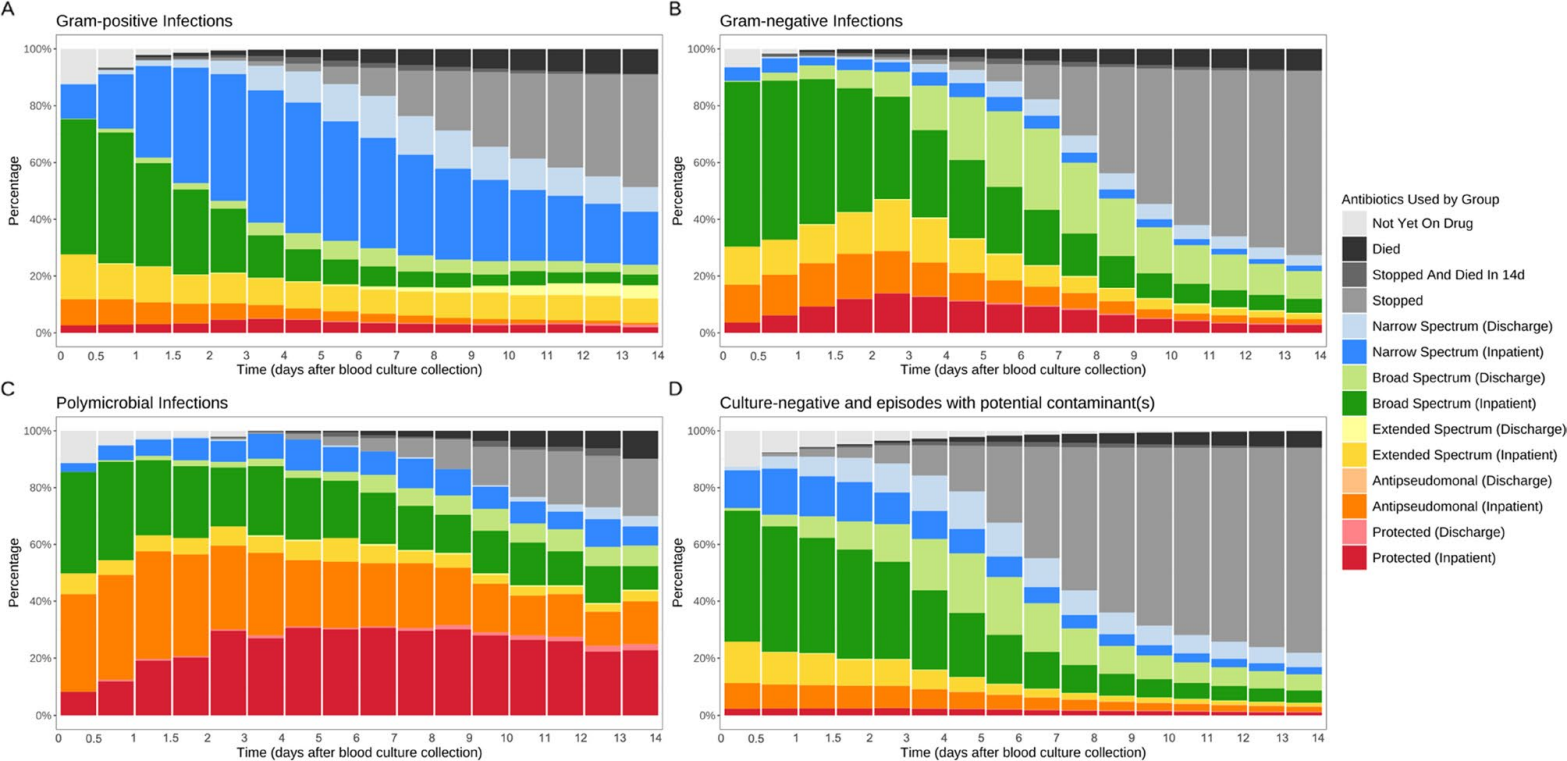
Trends in combined inpatient and discharge antibiotic use for suspected infection episodes within 14 days following index blood culture collection. 1,455 infections were with Gram-positive pathogens (**A**), 2,925 with Gram-negative pathogens (**B**), 193 with polymicrobial infections (**C**), and 46,389 with potential contaminants or culture-negative results (**D**)). Discharge antibiotic orders were in light colour, while inpatient orders were in dark colour

By day 7, 5.0% (73/1,455), 4.1% (120/2,925), and 1.6% (3/193) of patients with Gram-positive, Gram-negative, and polymicrobial infections, respectively, had died. In the remaining patients, there was notable de-escalation of treatment in those with Gram-positive bacterial infections, e.g., by day 7, 10.3% (142/1,382) had stopped antibiotics, and 56.6% (782/1382) had been switched to or continued on narrow-spectrum agents (Fig. 2A, Figure S4A). Switching was predominantly driven by the narrowing of treatment in confirmed infections with methicillin-susceptible *Staphylococcus aureus* and major Streptococcal pathogens. Comparatively, there was less de-escalation in Gram-negative infections by day 7; 12.6% (353/2805) had stopped antibiotics, and only 10.8% (303/2805) were being treated with narrow-spectrum antibiotics (Fig. 2B, Figure S4B). Similarly, antibiotics were only stopped in 4.7% (9/190) polymicrobial infections and narrow-spectrum treatment was given in 8.4% (16/190) by day 7. Additionally, 69.9% (135/193) of those with polymicrobial infections were still on antibiotic treatment after two weeks (vs. 51.3% for Gram-positive infections and 27.4% for Gram-negative infections) (Fig. 2C, Figure S4C).

Empirical broad-spectrum antibiotics were also widely used initially in suspected infections subsequently found to have negative blood cultures or potential contaminants, accounting for 83.0% (38,512/46,389) in the first three days. However, in most cases, treatment was stopped somewhat earlier, with 39.2% (18,187/45,122) stopping antibiotics by day 7 among surviving patients (Fig. 2D, Figure S4D).

In general, the most frequently prescribed empirical antibiotics were co-amoxiclav (oral and intravenous) and ceftriaxone (intravenous). Common escalations included transitions from oral co-amoxiclav or treatment suspension to intravenous co-amoxiclav. Conversely, the predominant de-escalations were from intravenous to oral co-amoxiclav (Table S3). Among 20,234 escalations (Table S4), resistance of an identified pathogen to the prior antibiotic regimen was documented in 6.2% of escalations involving Gram-positive pathogens, 19.0% for Gram-negative pathogens, and 26.1% for other pathogens or polymicrobial infections. Most de-escalations (77.0%) occurred in culture-negative episodes (Table S5). In episodes with a confirmed infection (17.6% of de-escalations), 83.3% and 8.8% of these de-escalations were to an antibiotic to which the identified pathogen was susceptible or resistant, respectively.

### Prescribing decisions following different CRP percentile changes

To assess how prescribing changed following each new CRP measurement, we considered pairs of CRP measurements taken on consecutive days. A total of 31,592 pairs of CRP measurements were taken on consecutive days during days 0–8 post-blood culture collection across 18,112 suspected infection episodes (see Figure S1 for study flow, Figure S2 for analysis illustration, and Table S6 for detailed results). The distribution of CRP percentile changes was clearly centred around no change in percentile; that is, these patients were recovering as expected, with their absolute CRP tracking the percentile that they were previously observed on (based on prior percentile development, Fig. 1 [5]). However, there were substantial minorities recovering both less well (positive percentile change, i.e. CRP increasing to a higher level or falling more slowly than expected given their starting value) and better (negative percentile change, i.e. CRP decreasing faster than expected given their starting value) than expected. Reflecting this distribution, 70.2% of cases (22,186/31,592) had a percentile change of < 15 in absolute magnitude, which we arbitrarily defined as recovering as expected. In 13.7% of cases (4,314/31,592), the CRP percentile decreased by > 15, suggesting a faster-than-expected recovery and conversely, 16.1% of cases (5,092/31,592) exhibited a sub-optimal recovery with a CRP percentile increase of > 15.

**Fig. 3:**
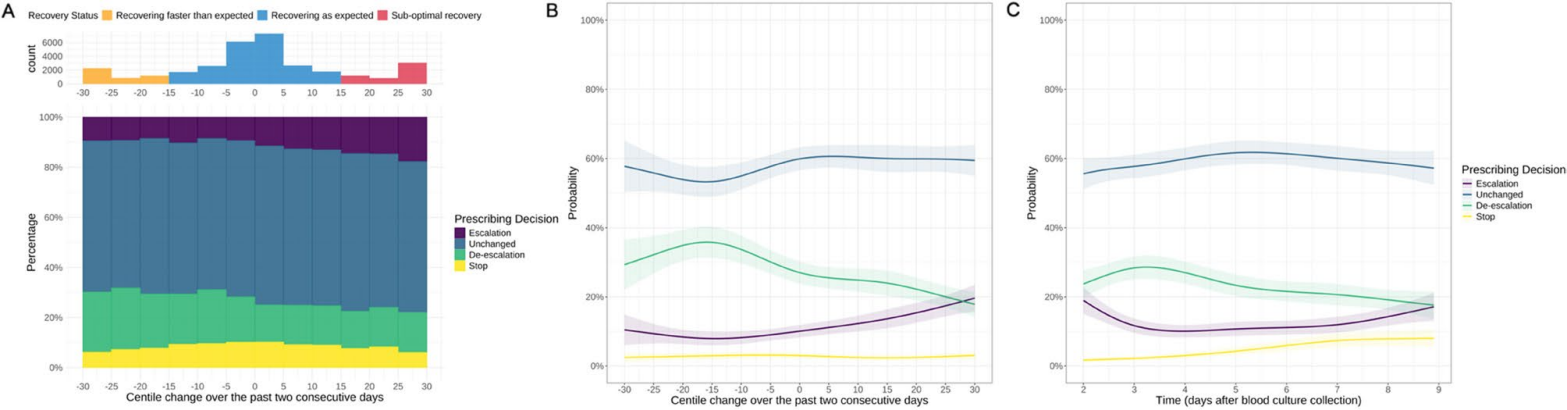
Crude percentage of prescribing decisions on days 2–8 after CRP percentile change over the two preceding consecutive days (**A**), predicted adjusted probabilities of prescribing decisions according to CRP percentile change over the two preceding consecutive days (**B**), and probabilities over time since index blood culture collection (**C**). Percentile change < − 15, − 15 to 15 and > 15 were arbitrarily defined as recovering faster than expected (orange), recovering as expected (blue) and sub-optimal recovery (red), respectively. See Figure S5 and Table S6 for the crude percentage split by culture-positive and culture-negative/contaminant episodes. Predictions are plotted using the reference values for other variables adjusted for in a multivariable model: prescribing day (time since index blood culture) = 4 (panel B only), percentile change = 0 (panel C only), age = 67 years, male, Charlson score = 2, Elixhauser score = 3, no renal dialysis, no diabetes, baseline NEWS = 3, absence of immunosuppression, no palliative care, community-onset, urinary source, and *E. coli* infection

Overall, across all pairs of CRP measurements, most antibiotics were unchanged in the 24 h following the second CRP measurement (61.8% [19,539/31,592]), where ‘unchanged’ included both no change in prescription and changes that resulted in the same antibiotic ranking, number of drugs, and route of administration (**Table S6**). More escalation occurred following sub-optimal recovery versus those recovering as expected (16.5% [838/5,092] vs. 10.7% [2,381/22,186], *p* < 0.001; 20.8% vs. 12.9% in culture-positive episodes, and 15.6% vs. 10.3% in culture-negative/contaminant episodes, *p* < 0.001; Table S6). Conversely, there was more de-escalation in cases recovering faster than expected (23.6% [1,016/4,314] vs. 17.2% [3,806/22,186], *p* < 0.001; 27.8% vs. 18.6% in culture-positive episodes, and 22.8% vs. 16.9% in culture-negative/contaminant episodes, *p* < 0.001; Table S6). Similar results were observed when comparing pairs of CRP from specific days, with higher rates of de-escalation and stopping, particularly after day 4, in patients recovering faster than expected or as expected (Figure S6), although some patients still receiving antibiotics escalated despite decreasing CRP centiles (i.e., improvement) and vice versa. The overall distribution of CRP percentile changes and the subsequent prescribing decisions (detailed in Table S6) are illustrated in Fig. 3A, with prescribing decisions further stratified by culture results in Figure S5.

After adjusting for patient and episode characteristics (including type of pathogen/contaminant/culture negative results), greater decreases in CRP centiles were independently associated with an increased likelihood of de-escalation, reaching ~ 35% if centiles decreased by 15–30 on day 4; while greater increases in CRP centiles were independently associated with a gradually increased likelihood of escalation, from 10 to 20%. However, the probability of antibiotics remaining unchanged was consistently 55–60% on day 4 following the index blood culture, regardless of CRP percentile changes over the past two days, likely reflecting the severity of the initial infection (visualised in Fig. 3B, based on the full adjusted model detailed in Table S7).

Independently, in these adjusted models, when patients were recovering as expected, i.e., tracking their CRP percentile, the probability of antibiotics remaining unchanged in spectrum (following our definition) was similarly high at 55–60% over 2 to 8 days from the initial blood culture (visualised in Fig. 3C, based on the full adjusted model detailed in **Table S7**). In these patients, the daily probability of stopping antibiotics was initially low but gradually increased to ~ 8% by day 8, whereas the probability of de-escalation peaked at 29% during days 3–4 before decreasing, and the probability of escalation dropped to 10% by day 4 before subsequently increasing (by definition in those still remaining on antibiotics).

Independently, patients on renal dialysis had a lower probability of de-escalation (Relative-Risk Ratio (RRR) = 0.79 [95%CI 0.66,0.93]) and stopping (RRR = 0.63 [0.50,0.79]) (**Table S7**). Immunosuppressed patients were also less likely to stop antibiotics (RRR = 0.79 [0.70,0.88]). Patients receiving palliative care were less likely to de-escalate (RRR = 0.60 [0.52,0.69]). For community-onset infections, the probability of either escalation or de-escalation was independently higher (RRR = 1.28 [1.18,1.39] for escalation; RRR = 1.80 [1.66,1.94] for de-escalation). Infections with *P. aeruginosa* and *S. aureus* were associated with a lower likelihood of stopping antibiotics compared to *E. coli* infections, likely reflecting more complex underlying clinical syndromes or specific guidance about their treatment duration. Potential contaminants and culture-negative results were associated with a higher likelihood of stopping antibiotics. Regarding infection sources, respiratory, multiple, and abdominal sources were associated with a higher probability of escalation compared to urinary tract infections.

### CRP percentile changes following different prescribing decisions

We also considered how CRP changed following a change in antibiotics. A total of 46,868 individual antibiotic prescription changes occurred within the 29,678 episodes eligible for this analysis (see Methods) to investigate the association between prescribing decisions and subsequent changes in CRP centiles (see Figure S1 for cohort derivation; Figure S3 illustrates this analytical approach). These prescription changes were followed by ≥ 1 CRP measurement from between 12 h before through 48 h after the change or the next prescription change, whichever occurred first (median 1 (IQR 1–1, range 2–8) measurements). Among these, 14,947 (31.9%) new prescriptions represented escalation decisions, 12,673 (27.0%) had no change in the spectrum of activity, 13,084 (27.9%) were de-escalation decisions, and 6,164 (13.2%) stopped antibiotics.

The average estimated CRP percentile changes following different prescribing decisions were relatively small and within the ± 15 percentiles threshold previously used [5] as consistent with the standard response (Figure S7). In general, CRP percentile levels were higher at the time of the prescribing decisions to escalate treatment and lower for prescribing decisions to stop antibiotics. Specifically, when prescribing decisions were made within the first four days after blood culture collection, CRP centiles tended to increase slightly over the next 48 h, regardless of whether the decision was to escalate, maintain, de-escalate, or stop antibiotic treatment. In contrast, for decisions to escalate, maintain, or de-escalate antibiotics made on or after day 5, CRP centiles remained stable or decreased over the following 48 h (interaction *p* < 0.0001). Notably, a patient recovering as expected with appropriate antibiotic management would be expected to maintain a constant CRP percentile.

### Associations between early changes in CRP centiles and mortality

**Fig. 4:**
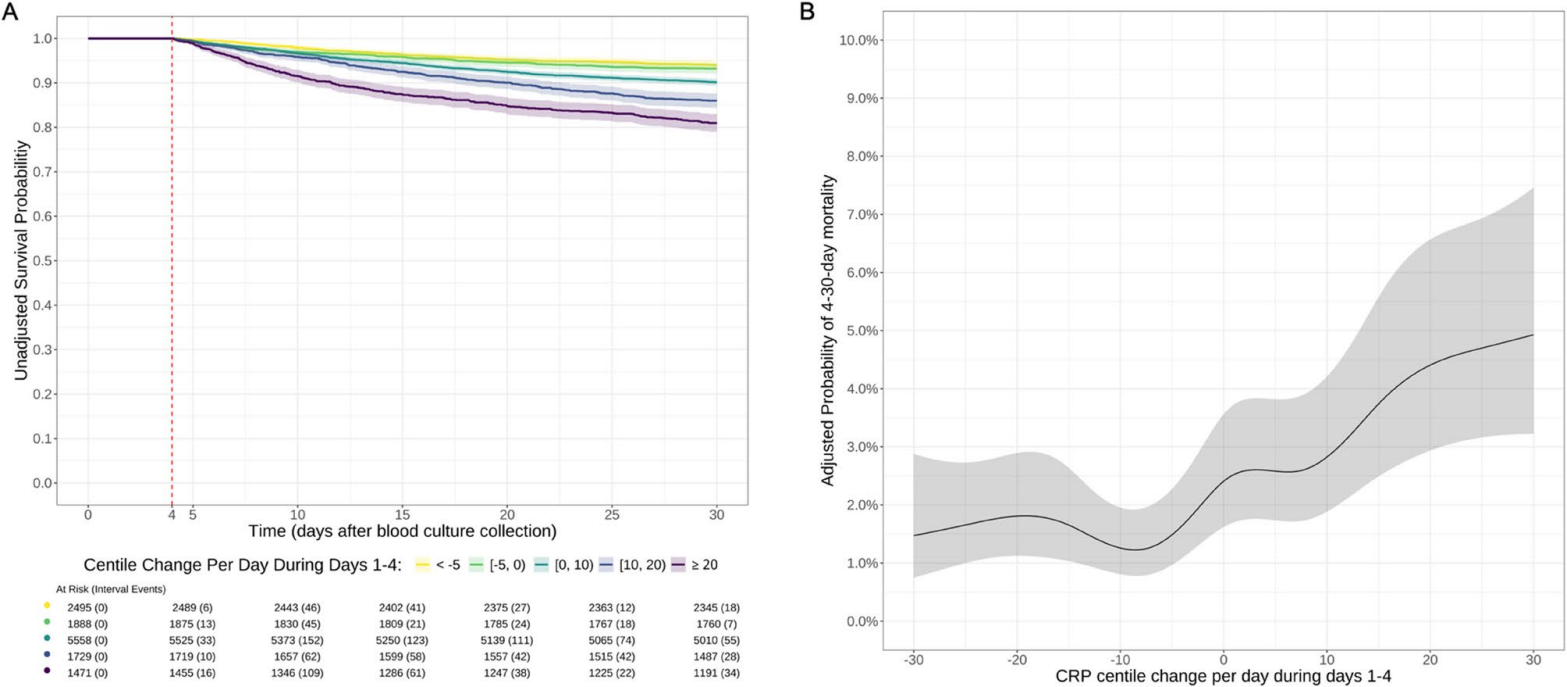
Unadjusted Kaplan-Meier curves and risk tables (**A**) and adjusted predicted probability of 5–30 day all-cause mortality (**B**) by mean CRP percentile change per day during days 1–4 The percentile change per day from panel A was divided into 5 categories: <−5, −5 to 0, 0 to 10, 10 to 20, and ≥ 20, based on the predicted probability from panel B. Predictions in panel B are plotted using the reference values for other variables in the adjusted model: age = 66 years, male, Charlson score = 2, Elixhauser score = 3, no renal dialysis, no diabetes, baseline NEWS = 3, absence of immunosuppression, no palliative care, community-onset, urinary source, and *E. coli* infection

Unadjusted Kaplan-Meier survival curves for mortality during 5–30 days after blood culture collection, stratified by individual patient-level mean CRP percentile change per day over days 1–4, showed that patients recovering better than expected, or as expected (with percentile changes of <−5 and [−5, 0) per day) consistently had the highest survival probabilities, ~ 94% by day 30 (Fig. 4A). These patients remained consistently separated during the day 5–30 follow-up from patients not recovering as expected (with rising percentile changes between days 1–4). Patients with the most marked sub-optimal recoveries (percentile changes of ≥ + 20/day) had the lowest survival probabilities, ~ 81% by day 30.

As there was no evidence of non-proportionality and in order to calculate predictive power using the area under the receiver operating curve (AUROC), we adjusted for potential confounders using a simpler logistic model. The probability of 5–30 day all-cause mortality increased non-linearly with higher daily CRP percentile changes during days 1–4 (Fig. 4B). At the reference category for other factors, the mortality risk was ~ 1.5% for a mean daily percentile change of <−5, increasing to ~ 2.5% for a mean daily percentile change of 0–10, and further increasing to ~ 4.7% for a mean daily percentile change of 20–30. Other factors associated with mortality included age (OR per 10 years older = 1.59 [95%CI 1.51,1.68], *p* < 0.001), Charlson score (OR = 1.15 per unit higher [1.07,1.23], *p* < 0.001), Elixhauser score (OR = 1.08 per unit higher [1.03,1.12], *p* = 0.001), diabetes mellitus (OR = 0.78 [0.65,0.92], *p* = 0.005), baseline NEWS score (OR = 1.11 per unit higher [1.08,1.13], *p* < 0.001), immunosuppression (OR = 1.36 [1.15,1.61], *p* = 0.001), and palliative care (OR = 13.6 [11.5,16.0], *p* < 0.001) (Table S8). Blood culture results and source also had strong associations with mortality risk, with the most substantial impacts from polymicrobial infections (OR = 2.69 vs. *E. coli* [1.33,5.27], *p* = 0.005) and central nervous system (CNS) infections (OR = 2.65 vs. urinary source [1.23,5.20], *p* = 0.008).

Compared to a logistic model adjusting for covariates only, most covariates showed small changes in their log OR after incorporating CRP percentile change (**Table S8**). However, several covariates exhibited more substantial changes, e.g., baseline NEWS (+ 20.0%), culture-negative infections (−46.7%), and CNS infections (+ 13.4%), indicating a more pronounced influence of adjusting for CRP percentile change on these factors.

AUROCs showed that the CRP percentile change over days 1–4 alone was only modestly predictive of day 5–30 mortality (AUROC = 0.65 [95%CI 0.62,0.68]), outperforming absolute CRP change (AUROC = 0.62 [0.58, 0.65]; *p* = 0.007) and comparable to log (i.e. relative) CRP change (AUROC = 0.65 [0.62, 0.68], *p* = 0.85) (Table 2). The covariates-only model achieved an AUROC of 0.84 [0.82,0.87]. The adjusted CRP percentile model achieved an AUROC of 0.86 [0.84,0.88], showing a small but statistically significant improvement over the covariates-only model (*p* < 0.0001).

**Table 2:** Predictive power of CRP percentile change, absolute CRP change, and log CRP change per day (days 1–4) for 5–30 day all-cause mortality, with and without covariate adjustment

	AUROC (95% CI)^1^	
Models	Unadjusted models	Adjusted models
Covariates only	–	0.84 (0.82–0.87)
CRP percentile change	0.65 (0.62–0.68)	0.86 (0.84–0.88)
Absolute CRP change	0.62 (0.58–0.65)	0.84 (0.82–0.87)
Log CRP change	0.65 (0.62–0.68)	0.85 (0.82–0.87)

^1^Area under the receiver operating characteristic curve (95% confidence interval)

## Discussion

We previously developed centile reference charts for expected CRP responses in suspected BSI (analogous to paediatric growth charts), which account for heterogeneity at initial presentation. Here, we investigated the relationship between changes in these CRP centiles, antibiotic prescribing, and patient outcomes in suspected infections. Notably, our study cohort comprised all inpatient episodes where clinicians requested collection of blood for culture, a pragmatic proxy for a clinically serious “suspected infection”. This ensured the inclusion of both culture-positive and culture-negative episodes, reflecting real-world practice and ensuring our findings are broadly applicable, given that we previously showed that over half of blood culture-negative cases have CRP responses indistinguishable from culture-positive patients. However, there could also be potential dilution bias due to non-infectious mimics. Whilst we found that CRP percentile changes were associated with both prior and subsequent prescribing decisions and with subsequent mortality, many observed effects were modest and prognostic, i.e. at the population level, rather than predictive, i.e. at the individual level, indicating the complexity of infection management and the multifactorial influences on patient outcomes.

The analysis of antibiotic prescribing demonstrated the expected initial reliance on broad-spectrum antibiotics within the first three days after the index blood culture across suspected infections regardless of (subsequent) blood culture result. This empirical approach reflects the urgency in managing suspected serious infections due to their high risk of morbidity and mortality [6]. For Gram-positive infections, there was a notable shift towards narrower-spectrum antibiotics within the first four days, with 42.9% of cases de-escalating, aligning with stewardship guidelines advocating for de-escalation to prevent the development of resistance and mitigate adverse drug effects [2]. Conversely, Gram-negative infections showed a slower decline in broad-spectrum antibiotic use, reflecting the complexity of some infections and the higher resistance potential associated with these pathogens [31]. Broad-spectrum antibiotics were also primarily used throughout the 14 days in polymicrobial infections, reflecting empirical treatment of predominantly intra-abdominal infection even when a specific species is isolated from blood.

Our detailed analysis of de-escalation events (occurring ≥ 48 h after index culture) in confirmed infections further elucidated these practices. A high proportion (83.3%) of such de-escalations appropriately targeted susceptible pathogens, supporting microbiology-guided stewardship. However, a notable minority (8.8%) of these de-escalations were to antibiotics to which an identified pathogen was resistant. This latter finding likely reflects several complex clinical scenarios rather than straightforward prescribing errors. For instance, some apparent de-escalations to a notionally ineffective agent may arise from short gaps in therapy during switches from one active drug to another, where a second background drug (inactive against the bloodstream pathogen but potentially targeting another concurrent infection) is continued; despite using a ‘window period’ to link short gaps in treatment, not all such instances may be perfectly captured. Other events may occur in polymicrobial infections where treatment for one component is completed (e.g., vancomycin stopped for *Enterococcus faecium* in an episode also involving *E. coli* treated with meropenem); the continued meropenem might then appear as ‘inactive treatment’ for the *E. faecium*, when the clinical intent was that this specific part of the therapy was complete. Nevertheless, a minority of these events likely do represent instances where the prescribed agent is genuinely reported as resistant to the primary pathogen, highlighting an area for ongoing stewardship focus. It is also important to note that the majority of all de-escalation events (77.0%) occurred in culture-negative cases, necessarily reflecting empirical decisions guided by clinical improvement.

Although a higher proportion of contaminant/culture-negative cases discontinued antibiotics, in those who continued antibiotics, broad-spectrum agents were disproportionately prescribed, particularly at discharge. This pattern underscores the inherent tension in management for suspected infection: the need for rapid, empirically broad coverage versus the imperative to minimise unnecessary antibiotic exposure [6]. Indeed, when treatment was escalated, documented pathogen resistance to prior therapy contributed to some decisions, particularly for Gram-negative (19.0%) and polymicrobial (26.1%) infections. However, most escalations (76.9%), predominantly in culture-negative episodes, were not associated with identified pathogen resistance, further underscoring the impact of clinical uncertainty in these decisions.

Changes in CRP centiles were associated with subsequent prescribing decisions in the next 24 h, albeit with small effect sizes. Prescribing decisions, which were assessed each day, mostly (61.8%) remained unchanged in terms of spectrum, following our definition, regardless of CRP percentile changes. This high rate of unchanged prescriptions could indicate that initial empiric therapy was appropriately selected and effective, or it might reflect clinician caution in altering treatment when patients show signs of recovery, even if biomarkers suggest potential for de-escalation. However, somewhat higher rates of escalation occurred in patients with sub-optimal recovery (16.5% vs. 10.7% in those recovering as expected), implying recognition of inadequate therapeutic response and a subsequent need for intervention. Conversely, faster than expected recovery was more frequently followed by de-escalation (23.6% vs. 17.2% in those recovering as expected). Similar trends were observed when the analysis was broken down into subgroups with only culture-positive and culture-negative/contaminant episodes. These findings complement existing studies that advocate for adaptable treatment strategies based on real-time clinical markers [6, 10], with CRP monitoring potentially contributing to personalised infection management. However, while these associations were identified at a population level, they were inconsistently observed for individual patients, with some still having antibiotics escalated despite improving CRP centiles and de-escalated despite increasing CRP centiles. Moreover, antibiotic prescribing decisions are influenced by a multitude of clinical factors beyond CRP levels, including vital signs, need for respiratory or cardiovascular support, changes in white blood cell count and differential, microbiological culture results, previous antibiotic exposure, radiologic findings, and the ability to achieve source control. The current study focused primarily on CRP percentile changes, which may oversimplify the complex decision-making process in clinical practice.

We also attempted to assess how CRP changed after a change in antibiotics. However, regardless of prescribing decisions, CRP centiles counter-intuitively tended to increase after any prescribing change, especially when this occurred in the first four days after blood culture collection. At least for those not escalating antibiotics, this pattern likely arises from more frequent CRP testing in those not recovering as expected, which then introduces a bias into our estimates.

For those whose treatment was escalated, the continued rise in CRP centiles could also reflect continued poor response to therapy, potentially due to factors such as unidentified antimicrobial resistance, inadequate antibiotic exposure at the site of infection, or, importantly, the lack of or delay in achieving adequate source control of the primary infection (e.g., drainage of an abscess, or removal of an infected medical device). Similar estimates in subsequent CRP centiles following de-escalation and no change in spectrum at all timepoints after blood culture collection are potentially indicative of appropriate de-escalation of treatment in the patients studied, with the selected narrower spectrum agent being equally effective against the causative pathogen(s) in terms of CRP response. Of note, this analysis was conditional on having at least one change in antibiotic prescription post-baseline with subsequent CRP measurements. Also, in most cases, only one CRP measurement was performed within 48 h after the prescribing decision, and the few instances of more frequent measurements may have preferentially occurred in deteriorating patients, with the same potential bias (i.e. upward trend) as repeated CRP measurements after antibiotic discontinuation. Addressing this issue fully would require sampling regardless of clinical progress, which is unfeasible on a large scale. Excluding those with only one measurement would lead to imprecise and biased estimates, particularly when the missing data mechanism is not completely at random [30]. Furthermore, the significant time-dependent confounding presents challenges in establishing causal relationships in this observational study, which is why we focussed on short-term changes before and after prescribing decisions and pairs of CRP measurements.

The association between individual patient-level mean CRP percentile changes over the first four days and subsequent mortality highlights the prognostic value of early CRP changes. AUROC analyses indicated that CRP percentile changes over days 1–4 modestly predicted day 5–30 mortality, outperforming absolute CRP changes and comparable to relative (i.e., log) CRP changes. Notably, adding early individual patient-level CRP percentile change into an adjusted logistic regression model decreased the log OR for culture-negative suspected infections by 47%, suggesting that CRP percentile change explained some variations in mortality risk previously attributed to culture-negative suspected infections, likely distinguishing more severe bacterial cases from less severe, and potentially non-bacterial, cases. This is consistent with our previous demonstration that 51.1% of culture-negative cases had increasing then decreasing CRP trajectories consistent with the standard response to infection (versus 64.5% of infections with an identified pathogen) [5]. In contrast, adding early individual patient-level CRP percentile change increased the log OR for CNS vs. urinary infections, suggesting that once CRP changes are accounted for, CNS infections are associated with higher mortality risk, likely due to their typically lower CRP response [5]. A similar effect was seen for baseline NEWS score. The minimal change in other effects, combined with the overall slight model performance improvement (AUROC from 0.84 to 0.86), indicates that while CRP refines risk prediction for certain subgroups, particularly in the context of suspected infections, the core mortality predictors remain unchanged.

This study has several limitations. First, our definition of a suspected infection episode relied on the clinical decision to obtain a blood culture. While this pragmatic approach mirrors routine care, it generates a heterogeneous cohort where fewer than half of the episodes are ultimately culture-positive for a pathogen. This heterogeneity may lead to misclassification and potentially dilute some biomarker associations. The observational design means we can only report associations, not infer causal effects of CRP changes on prescribing or vice versa, due to potential unmeasured confounding. Importantly, this study did not evaluate prescribing appropriateness against guidelines or antimicrobial susceptibility data, nor was it designed to assess the CRP centile charts as an antimicrobial stewardship (AMS) intervention. Our findings describe observed trends rather than auditing optimal practice or intervention efficacy, which would require different study designs.

Second, data on pre-admission antibiotic use were unavailable, and post-discharge durations were estimated from scheduled orders. Furthermore, we were not able to analyse several factors crucial to infection outcomes: detailed pathogen-specific drug resistance patterns (as individual combinations occurred in too few patients), appropriateness of initial antibiotic choice and dosing (not possible to assess from electronic health records), or precise timings of clinical suspicion and treatment initiation (clinical suspicion not recorded). These unanalysed elements, important determinants of mortality, could be sources of residual confounding. Similarly, a detailed comparative analysis of outcomes following de-escalation versus continued broad-spectrum therapy was beyond the scope of this specific study but remains clinically important.

Third, our antibiotic ranking system, while adapted from published work [26] and tailored to local practices, focused on the highest-ranked agent and categorised antibiotics into a fixed hierarchy; alternative classifications or more granular methods like the antibiotic spectrum index [32] might offer a more detailed quantitative assessment. The categorisation of antibiotics into any fixed hierarchy is a simplification, and the specific placement of certain agents may be debated, potentially yielding different results with alternative classifications. Interpreting CRP changes post-antibiotic adjustment is also complicated by CRP’s ~ 19-hour half-life and ~ 48-hour time to peak [33–35], meaning early CRP levels likely reflect prior therapy and infection dynamics. Our pragmatic analytical window (12 h before to 48 h after a prescribing decision, or until the next change) aimed to capture early CRP evolution in a dynamic setting, but means observed trajectories are a composite of these influences, ongoing infection severity, and potential testing biases. While a longer, fixed observation window might be theoretically preferable for observing a complete response to a new therapy in uncomplicated infections, this is challenging in real-world datasets with frequent interventions.

Antibiotic prescribing decisions are influenced by a multitude of clinical factors beyond CRP levels. The current study focused primarily on investigating associations with CRP percentile changes, which whilst tractable, inevitably oversimplifies the complex decision-making process in clinical practice. Future research could apply centile methods to faster-responding biomarkers (e.g., vital signs) and integrate multiple clinical factors to provide a more comprehensive assessment. Advanced modelling techniques, such as pharmacokinetic/pharmacodynamic modelling or time-series deep-learning approaches, might better disentangle biomarker dynamics and treatment decisions, although many of the challenges above will apply equally.

In summary, changes in CRP centiles appeared to have a modest association with antibiotic prescribing, predominantly at the population level, and early changes were strongly associated with subsequent mortality risk. These results indicate that whilst CRP monitoring can aid in antibiotic stewardship, its association with prescribing decisions is likely to be modest. Therefore, incorporating a broader spectrum of clinical factors is essential for optimising management for suspected infections.

## Electronic supplementary material

Supplementary Material 1

## Data Availability

The datasets analysed during the current study are not publicly available as they contain personal data but are available from the Infections in Oxfordshire Research Database (https://oxfordbrc.nihr.ac.uk/research-themes/modernising-medical-microbiology-and-big-infection-diagnostics/iord-about/), subject to an application and research proposal meeting the ethical and governance requirements of the Database. For further details on how to apply for access to the data and for a research proposal template please email iord@ndm.ox.ac.uk.
