# Supplementary material for: Interplay between C-reactive protein responses and antibiotic prescribing in people with suspected infection
Source: BMC Infect Dis. 2025 Aug 5;25:987. doi: 10.1186/s12879-025-11381-9 (PMC12326749; doi:10.1186/s12879-025-11381-9)
Supplement: Supplementary file 1 — Supplementary Material 1 [file 12879_2025_11381_MOESM1_ESM.docx]

# Interplay between C-reactive protein responses and antibiotic prescribing in people with suspected infection

## Supplementary Tables

Table S1. Classification and distribution of blood culture results.

| **Blood Culture Results** | N = 51,544 (100%)^1^ |
| --- | --- |
| Gram-positive Pathogens | |
| *Staphylococcus aureus* | 571 (1.1%) |
| Beta-Hemolytic Streptococci | 313 (0.6%) |
| *Enterococcus* sp. | 248 (0.5%) |
| *Streptococcus pneumoniae* | 224 (0.4%) |
| Other Pathogenic *Streptococcus* | 99 (0.2%) |
| Gram-negative Pathogens | |
| *Escherichia coli* | 1,937 (3.8%) |
| *Klebsiella* sp. | 400 (0.8%) |
| Other *Enterobacterales* | 282 (0.5%) |
| *Pseudomonas aeruginosa* | 212 (0.4%) |
| *Enterobacter* sp. | 94 (0.2%) |
| Other Pathogens | |
| Other | 379 (0.7%) |
| Polymicrobial | 193 (0.4%) |
| Anaerobes | 203 (0.4%) |
| Potential Contaminant(s) and Culture-negative | |
| Culture-negative | 43,743 (85%) |
| CoNS (contaminant) | 2,123 (4.1%) |
| Other Suspected Contaminants | 290 (0.6%) |
| Viridans and Other *Streptococcus* | 233 (0.5%) |
| ^1^n (%) |  |

Note: Other Pathogenic *Streptococcus* include *Streptococcus anginosus*, *Streptococcus gallolyticus*, *Streptococcus constellatus*, *Streptococcus intermedius*, *Streptococcus lutetiensis*, *Streptococcus bovis*. CoNS (contaminant) refers to Coagulase negative staphylococci. The "Other Suspected Contaminants" category was determined by a microbiologist includes organisms such as *Cutibacterium* spp. (formerly Propionibacterium), *Diphtheroids* (non-diphtheriae *Corynebacterium* species typically considered contaminants), *Micrococcus* spp., and *Bacillus* spp. (other than *B. anthracis*). The "Other" category under "Other Pathogens" comprises rarer pathogenic bacteria identified in blood cultures that did not belong to the more specifically defined pathogenic groups and were not categorised as contaminants (e.g, *Haemophilus influenzae*, *Acinetobacter lwoffii*, *Neisseria meningitidis*; also determined by a microbiologist).

Table S2. Ranking list of antibiotics.

| **1** | | **2** | **3** | **4** | **5** |
| --- | --- | --- | --- | --- | --- |
| Narrow Spectrum | Additions to any (or "narrow spectrum" if used as monotherapy) | Broad spectrum | Extended spectrum | Antipseudomonal | Protected |
| first/second generation cephalosporins^1^ | aminoglycosides^2^ | fluoroquinolones^3^ | third generation cephalosporins^4^ | antipseudomonal penicillin^5^ | antipseudomonal carbapenem^6^ |
| amoxicillin | clarithromycin | co-amoxiclav |  | ceftazidime | linezolid |
| metronidazole | clindamycin | co-trimoxazole |  | aztreonam | daptomycin |
| flucloxacillin | azithromycin |  |  |  | colistin |
| doxycycline | rrythromycin |  |  |  | tigecycline |
| nitrofurantoin | rifampicin |  |  |  | ceftazidime/avibactam |
| benzylpenicillin | vancomycin |  |  |  | ceftolozane/tazobactam |
| phenoxymethylpenicillin | teicoplanin |  |  |  | ertapenem |
| trimethoprim |  |  |  |  |  |
| fosfomycin |  |  |  |  |  |
| pivmecillinam |  |  |  |  |  |

^1^ First/Second generation cephalosporins: cefalexin, cefazolin, cefradine, cefuroxime

^2^ Aminoglycosides: amikacin, gentamicin, streptomycin, tobramycin

^3^ Fluoroquinolones: ciprofloxacin, ofloxacin, levofloxacin, moxifloxacin

^4^ Third generation cephalosporins: cefixime, cefotaxime, cefoxitin, ceftriaxone

^5^ Antipseudomonal penicillin: piperacillin/tazobactam, temocillin, piperacillin

^6^ Antipseudomonal carbapenem: imipenem, meropenem, imipenem/cilastatin

Note: Antibiotic agents used for suspected infections were ranked on a five-tiered scale with reference to the existing classification used by Moehring et al. (2021)^1^, which considered the agents’ spectrum of activity against bacterial pathogens, clinical importance as well as the priority for stewardship.

Table S3. Common antibiotics used for empirical, escalation and de-escalation treatments.

| **Common** **Empirical Agents (Days 0-2)** | **Antibiotic Rank** | **Usage in N (% of 51,544 episodes)** |
| --- | --- | --- |
| co-amoxiclav (IV)^1^ | Group 2 (Broad Spectrum) | 17,598 (34.1%) |
| co-amoxiclav (PO) | Group 2 (Broad Spectrum) | 8,711 (16.9%) |
| ceftriaxone (IV) | Group 3 (Extended Spectrum) | 6,432 (12.5%) |
| co-amoxiclav (IV) + gentamicin (IV) | Group 2 (Broad Spectrum) | 5,317 (10.3%) |
| piperacillin/tazobactam (IV) | Group 4 (Antipseudomonal) | 4,201 (8.2%) |
| **Common Escalation Events** **(Days 2-14)** | **Antibiotic Rank** | **Usage in N (% of** **20,234 escalation)** |
| stopped^2^ (N/A) → co-amoxiclav (IV) | N/A → Group 2 | 895 (4.4%) |
| co-amoxiclav (PO) → co-amoxiclav (IV) | Group 2 → Group 2 | 839 (4.1%) |
| co-amoxiclav (IV) → piperacillin/tazobactam (IV) | Group 2 → Group 4 | 480 (2.4%) |
| stopped^2^ (N/A) → co-amoxiclav (PO) | N/A → Group 2 | 449 (2.2%) |
| stopped^2^ (N/A) → piperacillin/tazobactam (IV) | N/A → Group 4 | 338 (1.7%) |
| **Common De-escalation Events (Days 2-14)** | **Antibiotic Rank** | **Usage in N (% of** **38,994 de-escalation events)** |
| co-amoxiclav (IV) → co-amoxiclav (PO) | Group 2 → Group 2 | 5,757 (12.9%) |
| piperacillin/tazobactam (IV) → co-amoxiclav (PO) | Group 4 → Group 2 | 1115 (2.9%) |
| clarithromycin (PO) + co-amoxiclav (PO) → clarithromycin (PO) | Group 2 → Group 1 | 854 (2.2%) |
| co-amoxiclav (IV) + gentamicin (IV) → co-amoxiclav (IV) | Group 2 → Group 2 | 849 (2.2%) |
| co-amoxiclav (IV) + metronidazole (PO) → co-amoxiclav (PO) + metronidazole (PO) | Group 2 → Group 2 | 814 (2.1%) |

^1^ Intravenous co-amoxiclav is recommended as empirical treatment for suspected infections at OUH.

^2^ Stopped means no antibiotics received for at least 48 hours, see Methods.
Note: Empirical treatments were defined as antibiotic agents administered within 48 hours after index blood culture collection. Escalation and de-escalation events were defined as those occurring more than 48 hours after index blood culture collection. IV=intravenous, PO=oral.

Table S4. Escalation events occurring 48h after the index blood culture (N=20,234) by blood culture result and susceptibility profile.

| **Blood Culture Result** | **Resistance-Related** | **Not Resistance-Related** | **Overall (% of N)** |
| --- | --- | --- | --- |
| Gram-positive Pathogens | 61 (6.2%) | 928 (93.8%) | 989 (4.9%) |
| Gram-negative Pathogens | 334 (19.0%) | 1,422 (81.0%) | 1,756 (8.7%) |
| Other Pathogens/Polymicrobial | 183 (26.1%) | 517 (73.9%) | 700 (3.5%) |
| Potential Contaminant(s) | NA | 1,230 (100.0%) | 1,230 (6.1%) |
| Culture-negative | NA | 15,559 (100.0%) | 15,559 (76.9%) |

Note: "Resistance-Related" refers to cases where pathogens showed resistance to antibiotics in the last prescription before escalation. Percentages in "Count (%) - Resistance-Related" and "Count (%) – Not Resistance-Related" columns represent row percentages, whilst figures in the "Overall (% of N)" column represent column percentages.

Table S5: Characteristics and context of de-escalation events (N = 38,994) occurring 48h after index blood culture.

| **Characteristic** | | **Count** | **Percentage** |
| --- | --- | --- | --- |
| **De-escalation Events by Overall Blood Culture Result** | | | |
|  | Confirmed Infection* | 6,866 | 17.6% |
|  | Culture-negative | 30,042 | 77.0% |
|  | Potential Contaminant(s) | 2,086 | 5.3% |
| **Microbiological Concordance of De-escalation in Confirmed Infections (N = 6,866)** | | | |
|  | Pathogen susceptible to the selected antibiotic | 5,720 | 83.3% |
|  | Pathogen resistant to the selected antibiotic | 607 | 8.8% |
|  | Pathogen susceptibility to the selected antibiotic unknown | 539 | 7.9% |
| **Top pathogen for De-escalations where the Pathogen was Resistant to the Selected Antibiotic (N = 607)** | | | |
|  | *E. coli* | 206 | 33.9% |
|  | Polymicrobial | 153 | 25.2% |
|  | *Enterococcus* sp. | 50 | 8.2% |
| **Top Antibiotics Selected in De-escalation where a Pathogen was Resistant (N = 607)** | | | |
|  | metronidazole | 186 | 30.6% |
|  | co-amoxiclav | 121 | 19.9% |
|  | vancomycin | 77 | 12.7% |

Note: * “Confirmed Infection” includes episodes with Gram-positive, Gram-negative, or Other/Polymicrobial pathogens identified from blood culture. Susceptibility was determined based on antimicrobial susceptibility testing results or, if unavailable for specific drugs, information on intrinsic resistance and antimicrobial activity from the Sanford Guide; cases with uncertainty were recorded as unknown.

Table S6. Prescribing decisions in the 24h following different categories of CRP percentile changes over the preceding two days.

| **Prescribing Decision** | **Recovering faster than expected**, N = 4,314^1^ | **Recovering as expected**, N = 22,186^1^ | | | **Sub-optimal recovery**, N = 5,092^1^ | | **Overall**, N = 31,592^1^ | **p-value**^2^ |
| --- | --- | --- | --- | --- | --- | --- | --- | --- |
| **All Episodes** | | | | | | **<0.001** | | |
| Escalation | 395 (9.2%) | 2,381 (10.7%) | | | 838 (16.5%) | | 3,614 (11.4%) |  |
| Unchanged | 2,609 (60.5%) | 13,823 (62.3%) | | | 3,107 (61.0%) | | 19,539 (61.8%) |  |
| De-escalation | 1,016 (23.6%) | 3,806 (17.2%) | | | 801 (15.7%) | | 5,623 (17.8%) |  |
| Stop | 294 (6.8%) | 2,176 (9.8%) | | | 346 (6.8%) | | 2,816 (8.9%) |  |
| **Culture-positive** **Episodes** | | | | **<0.001** | | | | |
| Escalation | 88 (13.8%) | 498 (12.9%) | | | 177 (20.8%) | | 763 (14.3%) |  |
| Unchanged | 353 (55.4%) | 2,492 (64.8%) | | | 505 (59.3%) | | 3,350 (62.8%) |  |
| De-escalation | 177 (27.8%) | 715 (18.6%) | | | 162 (19.0%) | | 1,054 (19.8%) |  |
| Stop | 19 (3.0%) | 142 (3.7%) | | | 8 (0.9%) | | 169 (3.2%) |  |
| **Culture-negative/Contaminant Episodes** | | | **<0.001** | | | | | |
| Escalation | 307 (8.3%) | 1,883 (10.3%) | | | 661 (15.6%) | | 2,851 (10.9%) |  |
| Unchanged | 2,256 (61.4%) | 11,331 (61.8%) | | | 2,602 (61.4%) | | 16,189 (61.7%) |  |
| De-escalation | 839 (22.8%) | 3,091 (16.9%) | | | 639 (15.1%) | | 4,569 (17.4%) |  |
| Stop | 275 (7.5%) | 2,034 (11.1%) | | | 338 (8.0%) | | 2,647 (10.1%) |  |
| ^1^n (%)  ^2^Pearson's Chi-squared test | | | | | | | | |

Note: Percentile changes were calculated from CRP measurements over two consecutive days and compared with prescribing decisions made in the 24h following the second CRP measurement. Percentile changes of <−15, −15 to 15 and >15 were considered as recovering faster than expected, recovering as expected and sub-optimal recovery, respectively. Presented pictorially in **Figure 3A** and **Figure S5**.

Table S7. Factors independently associated with different prescribing decisions following CRP percentile changes.

|  | **Escalation** | | | **De-escalation** | | | **Stop** | | |
| --- | --- | --- | --- | --- | --- | --- | --- | --- | --- |
| **Characteristic** | **RRR**^1^ | **95% CI**^1^ | **p-value** | **RRR**^1^ | **95% CI**^1^ | **p-value** | **RRR**^1^ | **95% CI**^1^ | **p-value** |
| Percentile change | See **Figure 3B** | — |  | See **Figure 3B** | — |  | See **Figure 3B** | — |  |
| Prescribing day | See **Figure 3C** | — |  | See **Figure 3C** | — |  | See **Figure 3C** | — |  |
| Age at admission (10 years) | 0.98 | 0.96, 1.00 | 0.072 | 0.99 | 0.97, 1.01 | 0.208 | **1.05** | **1.03, 1.08** | **<0.001** |
| Sex (male vs female) | 1.04 | 0.96, 1.11 | 0.344 | 1.06 | 0.99, 1.12 | 0.078 | **0.90** | **0.83, 0.98** | **0.013** |
| Charlson score | 1.03 | 0.99, 1.08 | 0.152 | 1.03 | 0.99, 1.07 | 0.175 | 1.03 | 0.98, 1.08 | 0.208 |
| Elixhauser score | **0.96** | **0.93, 0.98** | **0.001** | **0.94** | **0.92, 0.96** | **<0.001** | 0.99 | 0.96, 1.02 | 0.365 |
| Renal dialysis | 0.88 | 0.72, 1.07 | 0.193 | **0.79** | **0.66, 0.93** | **0.006** | **0.63** | **0.50, 0.79** | **<0.001** |
| Diabetes mellitus | 0.95 | 0.86, 1.05 | 0.330 | 0.97 | 0.89, 1.06 | 0.527 | 0.99 | 0.88, 1.10 | 0.798 |
| NEWS score (baseline) | 1.01 | 0.99, 1.02 | 0.271 | 1.01 | 1.00, 1.02 | 0.085 | 0.99 | 0.97, 1.00 | 0.162 |
| Immunosuppression | 1.02 | 0.93, 1.13 | 0.672 | **1.09** | **1.00, 1.18** | **0.039** | **0.79** | **0.70, 0.88** | **<0.001** |
| Palliative care | 1.07 | 0.94, 1.22 | 0.313 | **0.60** | **0.52, 0.69** | **<0.001** | 1.08 | 0.93, 1.26 | 0.323 |
| Community-onset | **1.28** | **1.18, 1.39** | **<0.001** | **1.80** | **1.66, 1.94** | **<0.001** | **0.87** | **0.80, 0.95** | **0.002** |
| Blood culture results |  |  |  |  |  |  |  |  |  |
| E. coli | — | — |  | — | — |  | — | — |  |
| Klebsiella sp. | 0.98 | 0.72, 1.32 | 0.879 | **1.32** | **1.02, 1.71** | **0.035** | 0.79 | 0.45, 1.39 | 0.418 |
| Other Enterobacterales | 0.96 | 0.67, 1.38 | 0.826 | 1.01 | 0.72, 1.40 | 0.963 | 0.48 | 0.21, 1.14 | 0.095 |
| Pseudomonas aeruginosa | 1.05 | 0.73, 1.50 | 0.795 | **0.67** | **0.46, 0.98** | **0.039** | **0.19** | **0.06, 0.61** | **0.005** |
| Enterobacter sp. | 0.73 | 0.40, 1.34 | 0.312 | 0.83 | 0.49, 1.40 | 0.477 | 0.61 | 0.24, 1.55 | 0.295 |
| Staphylococcus aureus | **0.70** | **0.53, 0.93** | **0.014** | 0.83 | 0.65, 1.06 | 0.134 | **0.22** | **0.10, 0.48** | **<0.001** |
| Beta-Hemolytic Streptococci | **0.61** | **0.39, 0.96** | **0.032** | **1.47** | **1.07, 2.01** | **0.016** | 1.09 | 0.49, 2.44 | 0.837 |
| Enterococcus sp. | **1.46** | **1.03, 2.05** | **0.032** | 1.26 | 0.90, 1.76 | 0.183 | 1.17 | 0.68, 2.01 | 0.566 |
| Streptococcus pneumoniae | 0.69 | 0.42, 1.11 | 0.127 | 1.40 | 0.98, 1.98 | 0.061 | 0.39 | 0.09, 1.60 | 0.189 |
| Other Pathogenic Streptococcus | **0.37** | **0.17, 0.82** | **0.014** | 0.77 | 0.42, 1.40 | 0.393 | 0.21 | 0.03, 1.51 | 0.120 |
| Other | 1.23 | 0.88, 1.71 | 0.222 | 1.10 | 0.80, 1.52 | 0.546 | 0.65 | 0.33, 1.29 | 0.217 |
| Polymicrobial | **1.46** | **1.07, 2.00** | **0.017** | 1.15 | 0.84, 1.58 | 0.379 | 0.55 | 0.29, 1.04 | 0.067 |
| Anaerobes | 0.77 | 0.48, 1.24 | 0.290 | 1.24 | 0.86, 1.80 | 0.247 | 0.88 | 0.39, 1.98 | 0.759 |
| Culture-negative | **0.66** | **0.57, 0.77** | **<0.001** | 0.89 | 0.78, 1.01 | 0.075 | **2.76** | **2.16, 3.53** | **<0.001** |
| CoNS (contaminant) | 0.90 | 0.73, 1.11 | 0.309 | 0.88 | 0.73, 1.07 | 0.215 | **2.84** | **2.12, 3.82** | **<0.001** |
| Other Suspected Contaminants | 1.03 | 0.63, 1.67 | 0.907 | 1.07 | 0.69, 1.66 | 0.758 | **2.74** | **1.53, 4.92** | **<0.001** |
| Viridans and Other Streptococcus | 0.86 | 0.54, 1.36 | 0.512 | 1.38 | 0.96, 1.99 | 0.079 | 1.13 | 0.58, 2.21 | 0.724 |
| Source of infection |  |  |  |  |  |  |  |  |  |
| Urinary | — | — |  | — | — |  | — | — |  |
| Unspecific | **0.67** | **0.57, 0.79** | **<0.001** | **0.73** | **0.65, 0.82** | **<0.001** | **1.38** | **1.18, 1.61** | **<0.001** |
| Respiratory | **1.26** | **1.10, 1.45** | **<0.001** | **0.83** | **0.75, 0.93** | **<0.001** | 0.90 | 0.77, 1.04 | 0.163 |
| Multiple sources | **1.87** | **1.63, 2.13** | **<0.001** | 1.05 | 0.95, 1.17 | 0.351 | **0.71** | **0.60, 0.83** | **<0.001** |
| Abdominal | **1.37** | **1.18, 1.59** | **<0.001** | **0.80** | **0.71, 0.90** | **<0.001** | 1.17 | 0.99, 1.37 | 0.062 |
| Skin, soft tissue, orthopaedic | 1.07 | 0.89, 1.29 | 0.483 | 0.89 | 0.78, 1.02 | 0.098 | **0.59** | **0.47, 0.73** | **<0.001** |
| CNS | 0.86 | 0.59, 1.28 | 0.463 | **0.55** | **0.39, 0.77** | **<0.001** | 1.10 | 0.77, 1.57 | 0.600 |
| Other | 1.06 | 0.82, 1.38 | 0.668 | 0.84 | 0.68, 1.04 | 0.116 | 0.78 | 0.56, 1.08 | 0.132 |
| ^1^RRR = Relative-Risk Ratio vs. Unchanged, CI = Confidence Interval | | | | | | | | | |

Note: The infection sources were categorised into respiratory, urinary, abdominal, skin/soft tissue/orthopaedic, central nervous system (CNS), other, multiple sources, and unspecific, based on the antimicrobial prescribing indications. The ‘unspecific’ category included episodes where the prescribing indication did not specify a particular infection source, while ‘other’ included less common sources not classified into the main categories.

Table S8. Factors independently associated with all-cause mortality over 5-30 days following index blood culture.

|  | **Covariates Only** | | | **Covariates + CRP percentile change** | | | **Change in log(OR) (%)** |
| --- | --- | --- | --- | --- | --- | --- | --- |
| **Characteristic** | **OR**^1^ | **95% CI**^1^ | **p-value** | **OR**^1^ | **95% CI**^1^ | **p-value** |  |
| Age at admission (10 years) | 1.59 | 1.51, 1.68 | **<0.001** | 1.58 | 1.50, 1.67 | **<0.001** | 0.0 |
| Sex (male vs female) | 1.13 | 0.99, 1.29 | 0.074 | 1.12 | 0.98, 1.28 | 0.10 | -8.3 |
| Charlson score | 1.15 | 1.07, 1.23 | **<0.001** | 1.16 | 1.08, 1.25 | **<0.001** | 7.1 |
| Elixhauser score | 1.08 | 1.03, 1.12 | **0.001** | 1.06 | 1.02, 1.11 | **0.008** | -14.3 |
| Renal dialysis | 0.78 | 0.49, 1.21 | 0.29 | 0.77 | 0.48, 1.18 | 0.25 | 12.5 |
| Diabetes mellitus | 0.78 | 0.65, 0.92 | **0.005** | 0.77 | 0.65, 0.92 | **0.004** | 4.0 |
| NEWS score (baseline) | 1.11 | 1.08, 1.13 | **<0.001** | 1.13 | 1.10, 1.15 | **<0.001** | 20.0 |
| Immunosuppression | 1.36 | 1.15, 1.61 | **<0.001** | 1.32 | 1.11, 1.57 | **0.001** | -9.7 |
| Palliative care | 13.6 | 11.5, 16.0 | **<0.001** | 12.7 | 10.7, 15.0 | **<0.001** | -3.8 |
| Community-onset | 0.98 | 0.85, 1.14 | 0.83 | 1.01 | 0.87, 1.18 | 0.89 | -150.0 |
| Blood culture result |  |  |  |  |  |  |  |
| E. coli | — | — |  | — | — |  |  |
| Klebsiella sp. | 1.26 | 0.61, 2.46 | 0.51 | 1.18 | 0.56, 2.31 | 0.65 | -30.4 |
| Other Enterobacterales | 2.01 | 0.99, 3.95 | **0.047** | 1.95 | 0.95, 3.85 | 0.060 | -4.3 |
| Pseudomonas aeruginosa | 0.73 | 0.28, 1.69 | 0.48 | 0.80 | 0.30, 1.88 | 0.62 | -28.1 |
| Enterobacter sp. | 1.07 | 0.23, 3.53 | 0.92 | 1.21 | 0.26, 3.95 | 0.78 | 171.4 |
| Staphylococcus aureus | 1.54 | 0.86, 2.70 | 0.14 | 1.53 | 0.86, 2.68 | 0.14 | 0.0 |
| Beta-Hemolytic Streptococci | 0.77 | 0.25, 1.90 | 0.60 | 0.75 | 0.25, 1.85 | 0.57 | 11.5 |
| Enterococcus sp. | 1.64 | 0.78, 3.29 | 0.18 | 1.43 | 0.67, 2.89 | 0.33 | -28.0 |
| Streptococcus pneumoniae | 0.57 | 0.20, 1.42 | 0.27 | 0.60 | 0.20, 1.52 | 0.32 | -9.1 |
| Other Pathogenic Streptococcus | 1.10 | 0.33, 3.38 | 0.87 | 0.92 | 0.27, 2.82 | 0.89 | -180.0 |
| Other | 2.51 | 1.26, 4.79 | **0.007** | 2.17 | 1.09, 4.13 | **0.022** | -15.2 |
| Polymicrobial | 2.69 | 1.33, 5.27 | **0.005** | 2.44 | 1.20, 4.79 | **0.011** | -10.1 |
| Anaerobes | 1.71 | 0.61, 4.24 | 0.28 | 1.58 | 0.56, 3.97 | 0.36 | -13.2 |
| Culture-negative | 1.35 | 0.99, 1.87 | 0.067 | 1.18 | 0.86, 1.63 | 0.32 | -46.7 |
| CoNS (contaminant) | 1.30 | 0.85, 2.00 | 0.23 | 1.13 | 0.73, 1.74 | 0.58 | -53.8 |
| Other Suspected Contaminants | 0.92 | 0.21, 2.83 | 0.89 | 0.84 | 0.18, 2.67 | 0.80 | 88.9 |
| Viridans and Other Streptococcus | 0.70 | 0.18, 2.04 | 0.56 | 0.55 | 0.14, 1.63 | 0.34 | 66.7 |
| Source of infection |  |  |  |  |  |  |  |
| Urinary | — | — |  | — | — |  |  |
| Unspecific | 1.27 | 0.96, 1.70 | 0.10 | 1.27 | 0.95, 1.69 | 0.11 | 0.0 |
| Respiratory | 2.30 | 1.80, 2.95 | **<0.001** | 2.26 | 1.77, 2.91 | **<0.001** | -1.2 |
| Multiple sources | 1.75 | 1.36, 2.26 | **<0.001** | 1.72 | 1.33, 2.24 | **<0.001** | -3.6 |
| Abdominal | 1.13 | 0.83, 1.54 | 0.43 | 1.16 | 0.85, 1.59 | 0.34 | 25.0 |
| Skin, soft tissue, orthopedic | 0.67 | 0.43, 1.03 | 0.073 | 0.68 | 0.43, 1.05 | 0.087 | -2.5 |
| CNS | 2.65 | 1.23, 5.20 | **0.008** | 2.93 | 1.36, 5.77 | **0.003** | 13.4 |
| Other | 1.29 | 0.71, 2.24 | 0.38 | 1.26 | 0.70, 2.18 | 0.43 | -11.5 |
| ^1^OR = Odds Ratio, CI = Confidence Interval | | | | | | |  |

Note: Mean CRP percentile change was calculated for each individual patient using ordinary least squares regression on values from day 1 to day 4, see **Methods**. Change in log(OR)% = ((log(OR) with CRP percentile change- log(OR) covariates only) / log(OR) covariates only) × 100, used to quantify the percentage change in the association of a covariate with the outcome after adjusting for CRP percentile change.

## Supplementary Figures


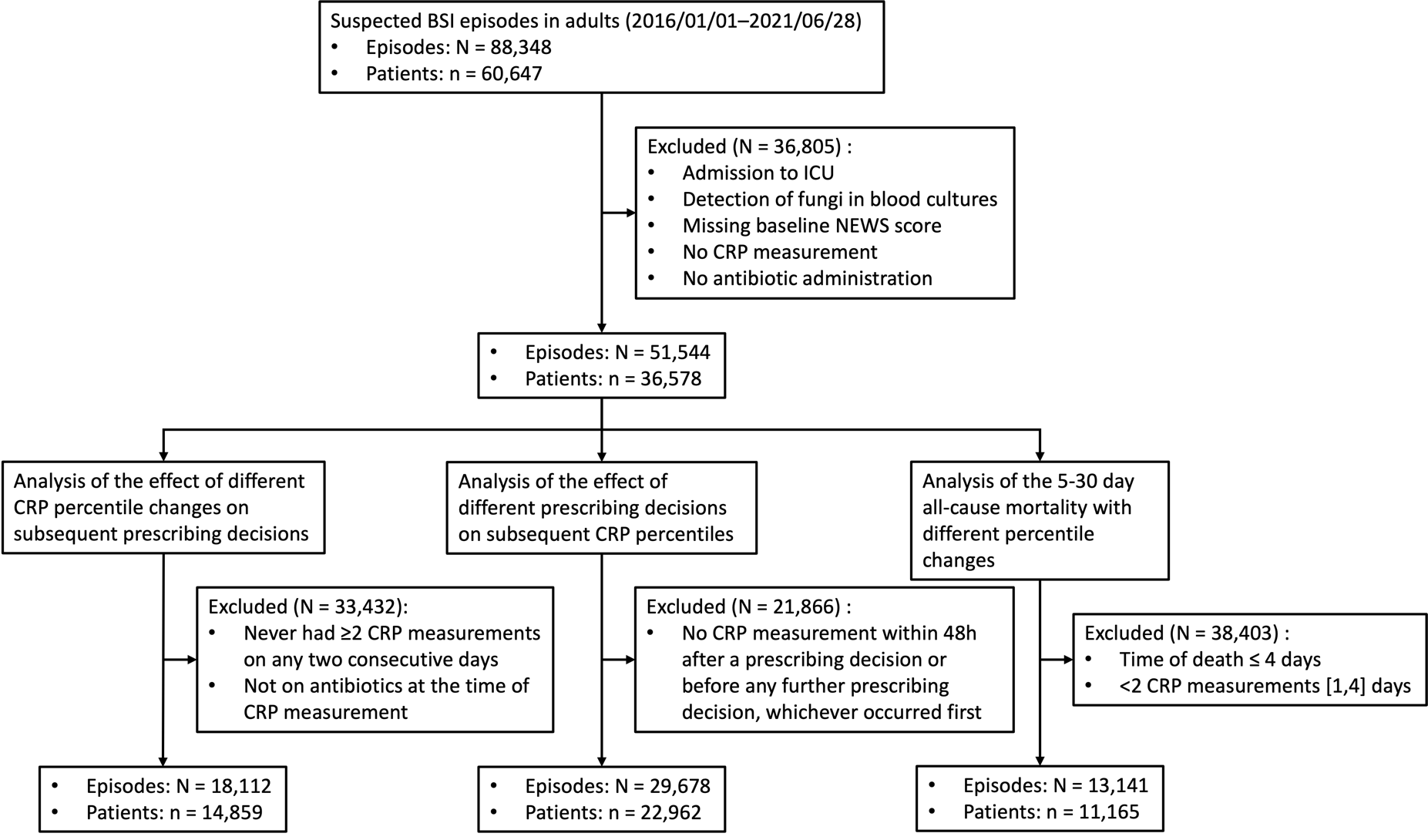


Figure S1. Flowchart to identify study population for different analyses.


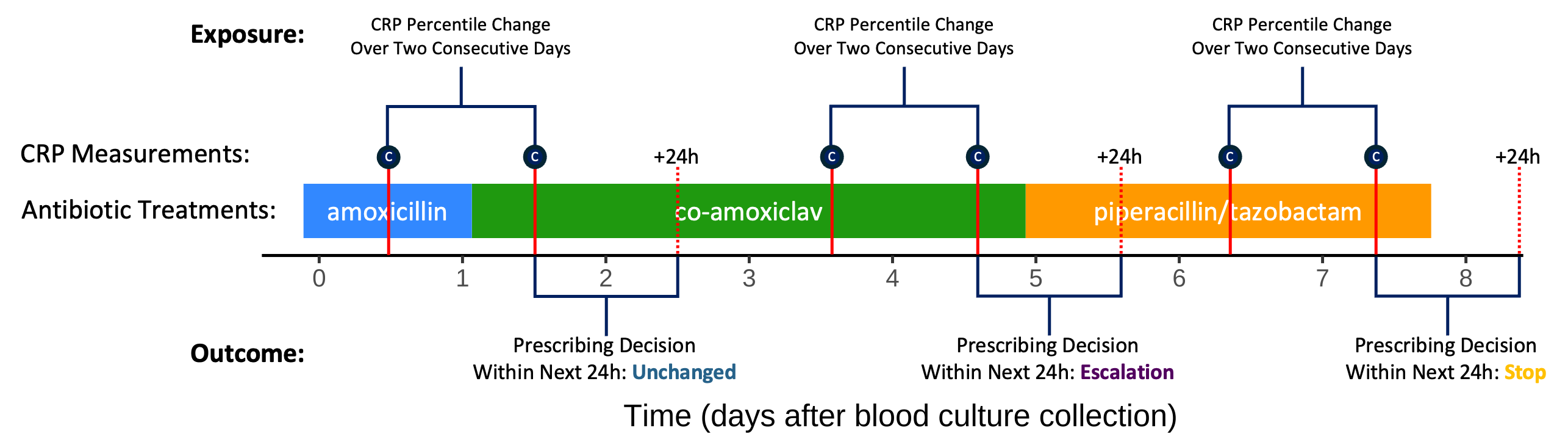


Figure S2. Illustrative diagram of analysis of the association between changes in CRP centiles and prescribing decisions.


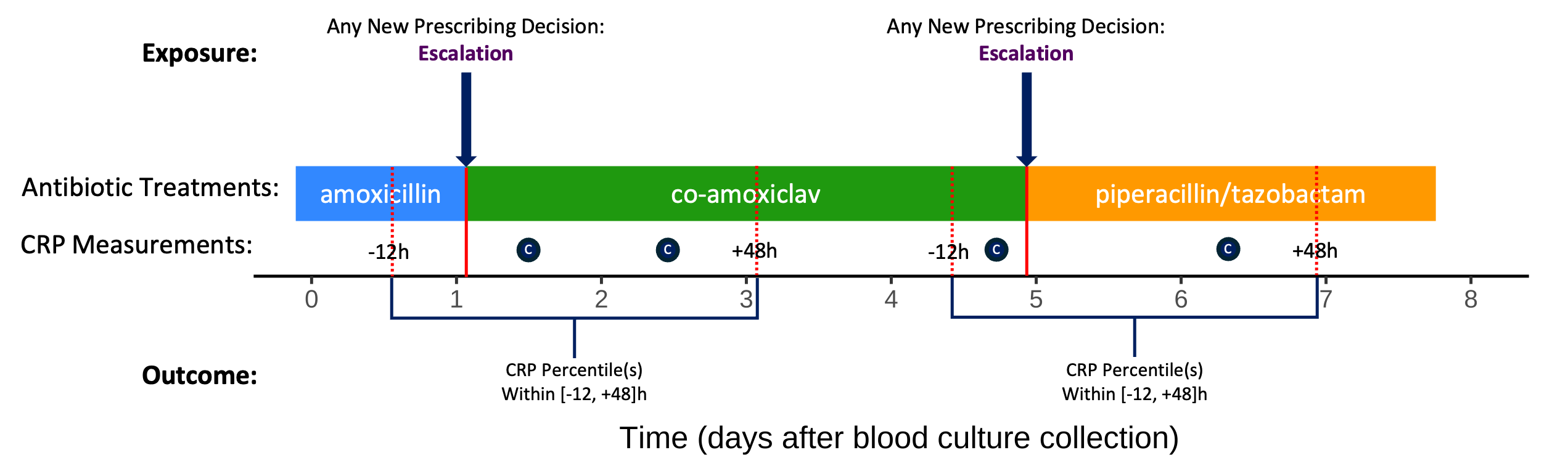


Figure S3. Illustrative diagram of analysis of the association between prescribing decision and CRP percentile changes.


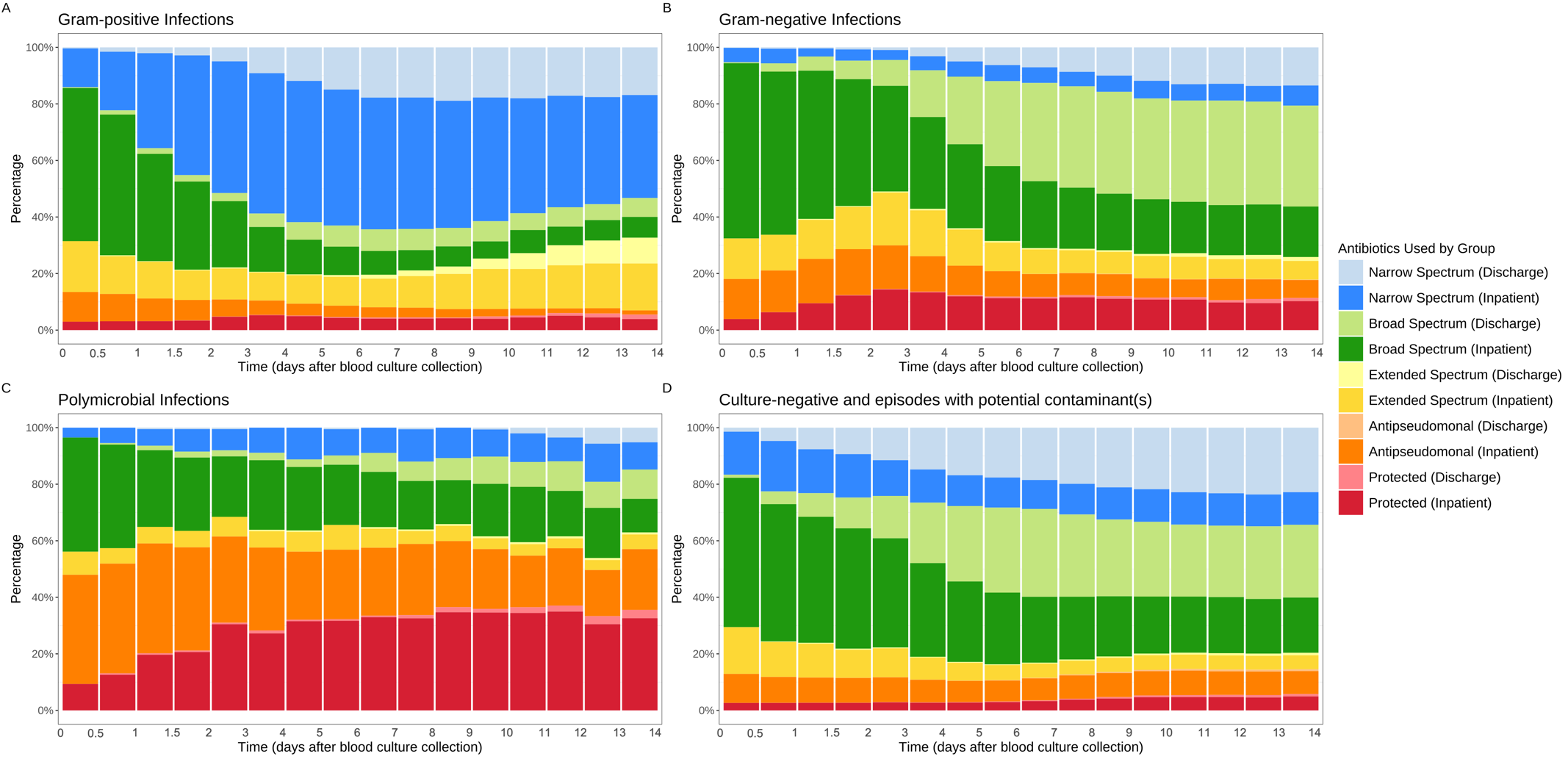


Figure S4. Trends in combined inpatient and discharge antibiotic use for suspected infection episodes where the patient was still on antibiotics, within 14 days following index blood culture collection.

Gram-positive pathogens in panel A, Gram-negative pathogens in panel B, polymicrobial infections in panel C, and potential contaminants or culture-negative results in panel D. Discharge antibiotic orders were in light colour, while inpatient orders were in dark colour.


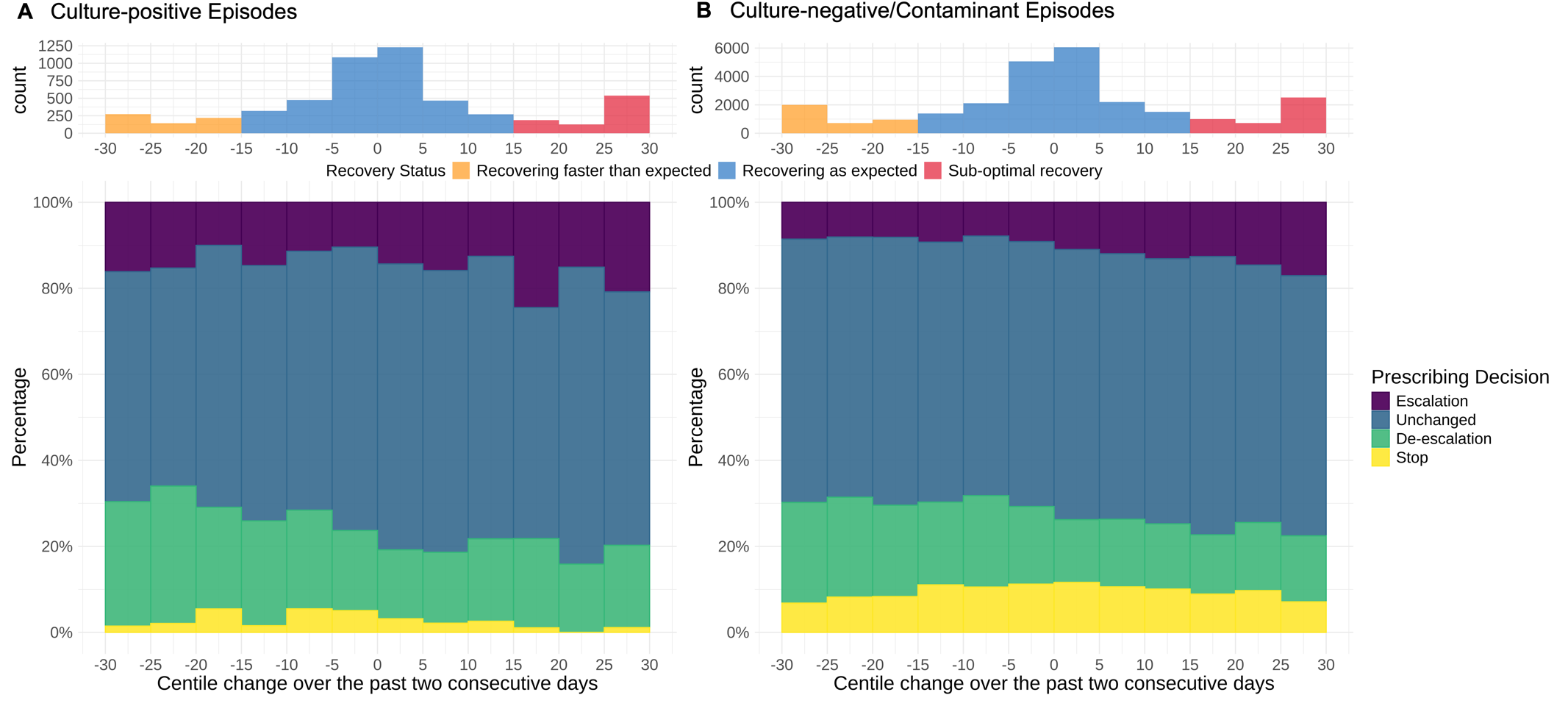


Figure S5. Crude percentage of prescribing decisions on days 2-8 after CRP percentile change over the two preceding consecutive days in culture-positive episodes (A) and culture-negative/contaminant episodes (B).

Percentile change <−15, −15 to 15 and >15 were arbitrarily defined as recovering faster than expected (orange), recovering as expected (blue) and sub-optimal recovery (red), respectively. See **Figure 3A** for the crude percentage in all episodes.


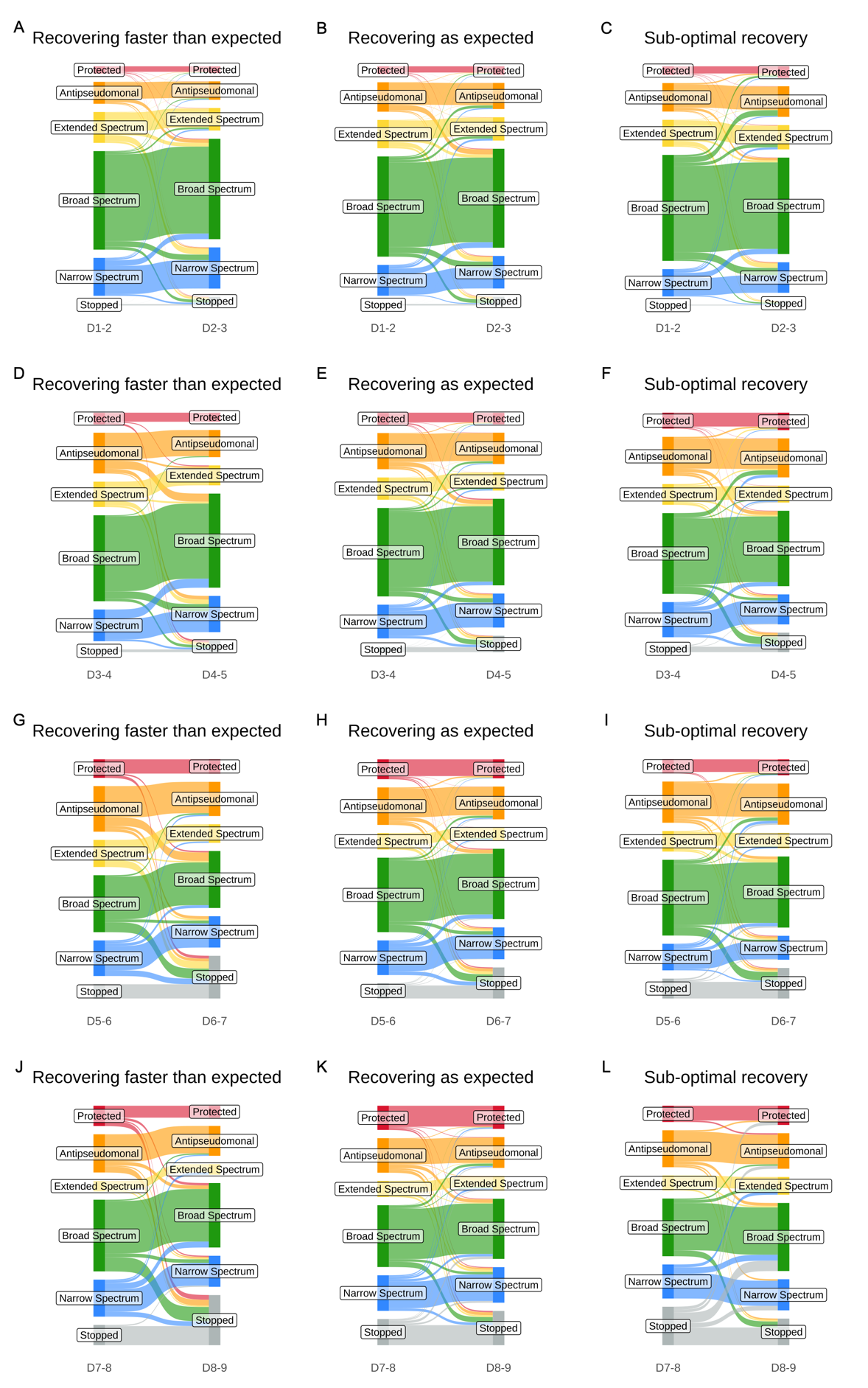


Figure S6. Distribution of change in antibiotic groups from day 1 to day 2, day 3 to day 4, day 5 to day 6, and day 7 to day 8 following different CRP percentile changes over two consecutive days.

Percentile changes of <−15, −15 to 15 and >15 were arbitrarily defined as recovering faster than expected, recovering as expected and sub-optimal recovery, respectively.


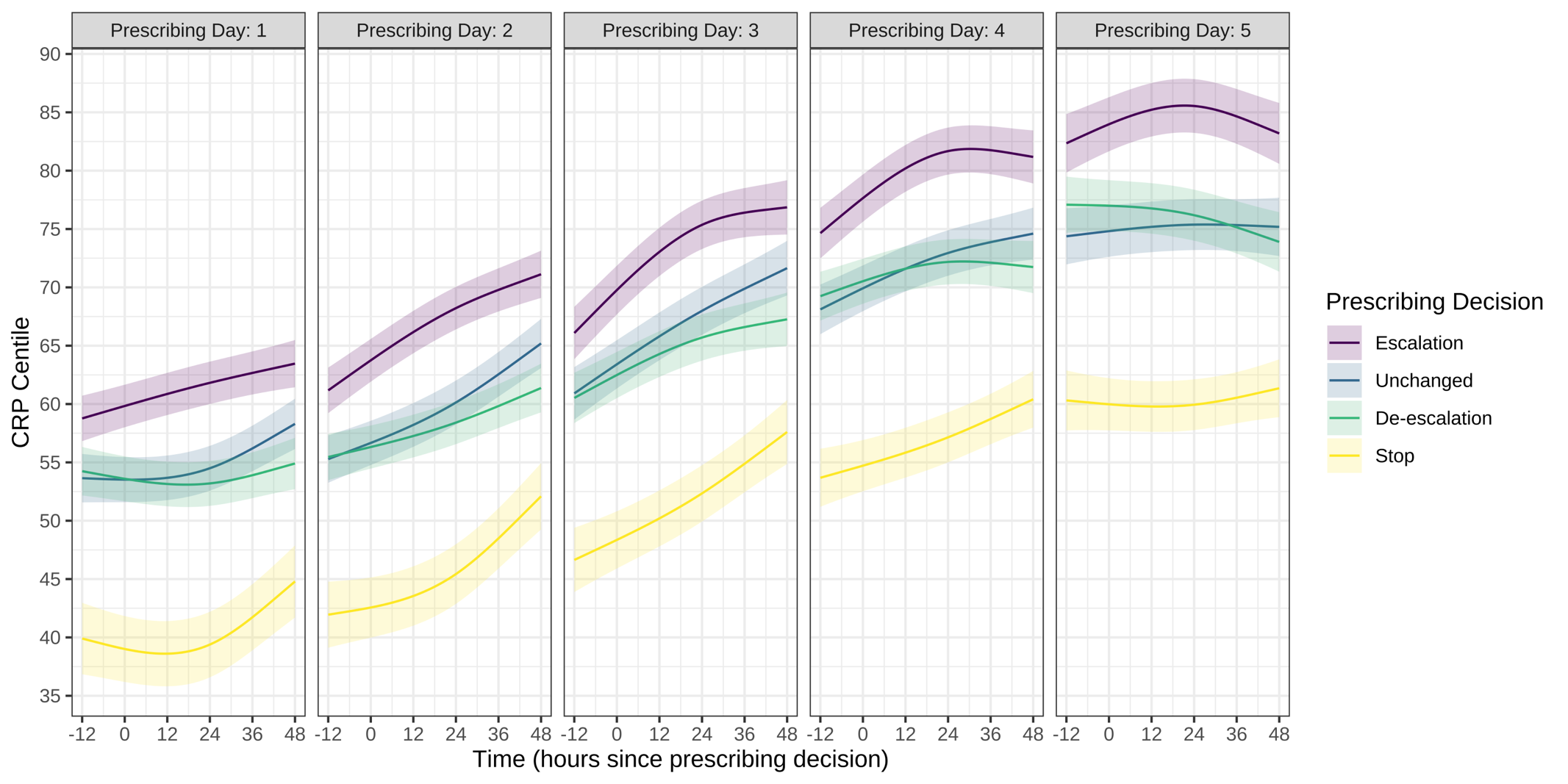


Figure S7. Predicted CRP percentile trajectories over the next 48 hours following different prescribing decisions on days 1, 2, 3, 4 and 5 since blood culture collection (prescribing day).

Predictions are plotted using the reference values for other variables adjusted for in a multivariable model: age = 67 years, male, Charlson score = 2, Elixhauser score = 3, no renal dialysis, no diabetes, baseline NEWS = 3, absence of immunosuppression, no palliative care, community-onset, urinary source, and *E. coli* infection.

## Reference

1. Moehring, R. W. *et al.* Development of an Electronic Definition for De-escalation of Antibiotics in Hospitalized Patients. *Clinical Infectious Diseases* **73**, e4507–e4514 (2021).
